# Exploring the repertoire of rhomboid proteases in *Cryptosporidium parvum* parasite: phylogenesis, structural motifs, and cellular localization in sporozoite cells

**DOI:** 10.3389/fcimb.2026.1733450

**Published:** 2026-04-07

**Authors:** Ilaria Vanni, Elisabetta Pizzi, Lucia Bertuccini, Alessandra Ludovisi, Fabio Tosini

**Affiliations:** 1 Department of Food Safety, Nutrition and Veterinary Public Health, Istituto Superiore di Sanità, Rome, Italy; 2 Core Facilities, Istituto Superiore di Sanità, Rome, Italy; 3 Department of Infectious Diseases, Istituto Superiore di Sanità, Rome, Italy

**Keywords:** *Cryptosporidium*, excystation, PARL rhomboid, phylogenesis, rhomboid, sporozoite, Sushi domain, thrombospondin-related anonymous proteins (TRAP)

## Abstract

*Cryptosporidium parvum* is an apicomplexan parasite and an important pathogen of mammals and humans, which can be infected by zoonotic transmission or directly by human-to-human contact. This parasite attacks the small intestine, and the main symptom is a watery diarrhea that can be particularly severe in newborns and deadly in immunodeficient subjects. Rhomboids are ubiquitous proteases embedded in cell membranes that act by cleaving other membrane proteins in or near their transmembrane domains. Apicomplexan rhomboids play an important role in approaching and invading the host cell. This study analyzed the phylogenetic origin, the structural motifs, and the subcellular localization of *C. parvum* rhomboids. Altogether, *C. parvum* possesses three rhomboids, namely, CpRom1, CpRom2, and CpRom3. The similarity search in *Cryptosporidium* genus revealed that *C. parvum* and other “intestinal” species lack a PARL-like rhomboid whereas this type of mitochondrial rhomboid was present in “gastric” species like *Cryptosporidium muris* and *Cryptosporidium andersoni.* At the genome level, this was revealed by a precise excision of the PARL-like gene in intestinal species whereas the rest of chromosomal synteny was well conserved among the *Cryptosporidium* species. The analysis of the structural domains revealed that *C. parvum* rhomboids can be classified as mixed secretases, and the comparison with orthologs from *Toxoplasma gondii* and *Plasmodium falciparum* showed that *C. parvum* rhomboids can be distinguished in two separate clusters based on similarities at the level of the catalytic sites. The three rhomboids were expressed simultaneously in the invasive stage of sporozoite, but each of them had a different spatial distribution. Indeed, CpRom1 had a dual localization: this rhomboid was internal at the apical complex, and it was also accumulated at the posterior pole of the sporozoite. Otherwise, CpRom2 was prevalently contained in the apical complex, and a point of accumulation was on the surface of the apical end. Differently from CpRom1 and CpRom2, CpRom3 is distributed along the entire surface of sporozoites. Finally, we listed 10 membrane proteins as candidate substrates for the *C. parvum* rhomboids based on the similarities with some proven substrates of apicomplexan rhomboids and the copresence in subcellular structures with the three rhomboids.

## Introduction

The genus *Cryptosporidium*, which belongs to the phylum Apicomplexa, is composed of many species of obligate parasites that in turn affect many species of vertebrates. *Cryptosporidium parvum* is the prevalent cause of cryptosporidiosis, a gastroenteric disease characterized by acute diarrhea, among many mammalian species, particularly ruminants and humans (Ryan et al., 2021). Indeed, *C. parvum* is often associated with human outbreaks caused by zoonotic transmission from livestock and wild ruminants (Helmy and Hafez, 2022). It is also considered a dangerous pathogen for immunodeficient subjects, in which the infection results in a watery diarrhea that can last for weeks, leading to severe and sometimes deadly dehydration (Balendran et al., 2024). Moreover, *C. parvum* and the strictly related *Cryptosporidium hominis* are the leading causes of mild and severe diarrheal cases among neonates and very young children in developing countries, accounting for thousands of deaths every year (Khalil et al., 2018). Despite its relevance for animal and human health, there are no effective treatments for this pathogen, and a viable vaccine is still to be conceived.

Rhomboids are serine proteases provided with various transmembrane (TM) domains, a bundle composed of six to seven TM helices spanning the lipidic bilayer. The rhomboid family has numerous members that are present in all kingdoms of life, from bacteria to mammals.

As proteases, rhomboids cleave other TM proteins internally or near their TM domain, producing two separate segments of the cleaved protein (Strisovsky, 2013). A mechanistic model of action of rhomboids is based on a three-dimensional reconstruction that shows that the TMs, those that compose the entire rhomboid domain, are organized like a sort of barrel embedded in the lipidic bilayer, with a slot on a side to allow the entry of the TM domains of the target protein in the middle of the proteolytic domain, where the cleavage occurs. This process will thus produce an internal segment that will remain toward the inner side of the membrane, whereas the other protein portion will be released from the outer membrane (Strisovsky, 2013). These proteases bear a characteristic dyad as a catalytic site consisting of a short consensus at TM4, namely, GXSX, where X can represent various amino acids (aa), and an essential histidine placed at TM6. These amino acid positions are conserved in all rhomboids with proteolytic activity and allow distinguishing between rhomboids and phylogenetically related but inactive pseudo-rhomboids (Tichá et al., 2018). However, other conserved amino acids flank these critical residues, so that, together with the number and the disposition of the TM domains, rhomboids can be further distinguished in different subclasses, such as secretase type A, type B, and mixed secretases (Lastun et al., 2016). A further subclass is represented by PARL, which are specifically linked to the inner mitochondrial membrane and that can be distinguished at the structural level for the presence of an additional TM domain at the N-terminus, thus implying that the catalytic dyad is moved from TM domains 4 and 6 to TM5 and TM7 (Lysyk et al., 2020). Obviously, rhomboids require to be allocated in a lipid bilayer to exert their proteolytic action, and the cleavage mediated by rhomboids is an irreversible step in important biological processes. Thus, rhomboids mediate EGFR signaling in *Drosophila* through the proteolysis of Spitz; the quorum sensing in bacteria like *Providencia stuartii*; the immune evasion in *Entamoeba histolytica*; and the homeostasis of mitochondrial membrane in man and other organisms (Urban and Dickey, 2011). However, thanks to the sequencing of myriads of genomes, we now know thousands of rhomboid homologs, although the function of most of them remains unknown.

The rhomboid superfamily also includes pseudoproteases, which conserve the overall structure of rhomboids but lack the enzymatic proteolytic activity. Among these, we find the iRhoms, which are considered regulators of stability and trafficking of other membrane proteins as observed for human iRom2 (Dulloo et al., 2019), and the so-called derlins, which are specialized types of pseudoproteases that reside in the endoplasmic reticulum (ER) and are involved in the ER-associated degradation (ERAD) pathway that contributes to the degradation of defective proteins (Lemberg and Strisovsky, 2021). Derlins have also been identified in the apicoplast, which is an exclusive organelle of Apicomplexa, in *Plasmodium falciparum*, and in other apicomplexans (Gallagher et al., 2011).

In general terms, apicomplexan parasites like *Toxoplasma gondii* and *P. falciparum* have a greater number of functional rhomboids if compared to mammals: seven rhomboids in *P. falciparum* and six in *T. gondii* with respect to the five identified in humans (Bergbold and Lemberg, 2013). Indeed, rhomboids in Apicomplexa play crucial roles during the invasion of the host cell. Thus, as demonstrated in *T. gondii* (Carruthers, 2006) as well as in *P. falciparum*, some rhomboids act by progressively cutting away adhesins (i.e., adhesive proteins distributed on the parasite surface) from the parasite external membrane to allow the gliding in the extracellular matrix and to favor the entry in the host cell (Sibley, 2013).

Some years ago, while looking for specific antigens of *C. parvum* sporozoites, we identified a novel rhomboid owing to the immunogenicity of a short peptide at its N-terminus, which we referred to as CpRom; this rhomboid, composed of 990 amino acids, is the largest rhomboid ever described (Trasarti et al., 2007). More recently, investigating the functional role of this protein, we have fortuitously found out that this rhomboid, now named CpRom1, is associated with the membrane of large extracellular vesicles released by the excysted sporozoites (Bertuccini et al., 2024). A quick search by similarity with rhomboids reveals that the *C. parvum* genome encodes for three functional rhomboids in total, but a comprehensive analysis of these rhomboids is still lacking. The aim of this study is to compare these rhomboids with the other apicomplexan rhomboids in terms of phylogenetic origin and similarity as well as of their expression in the invasive stage of sporozoites and subcellular localization. Furthermore, the high similarities of some *C. parvum* proteins with apicomplexan adhesins allowed the identification of plausible cleavage substrates for the three *C. parvum* rhomboids.

## Materials and methods

### General microbiological and DNA techniques

The recombinant and microbiological techniques, media, the preparation of plasmid DNA, and the isolation of restricted fragments all followed standard procedures (Sambrook et al., 1989). DNA sequencing was performed using Sanger’s procedure (Sanger et al., 1977).

### Sporozoite purification

Fresh *C. parvum* oocysts (Iowa strain) were supplied by Bunch Grass Farm (Deary, Idaho USA), stored at 4 °C in PBS with penicillin (1,000 U.I./mL) and streptomycin (1 mg/mL). Aliquots of 1 × 10^5^ per milliliter for microscopy or 1 × 10^7^ oocysts per milliliter for total lysate were used in the following experiments. The excystation procedure was performed as previously described (Bertuccini et al., 2024).

Excystation mixtures were sampled at various times after the induction, and excystation stopped in ice.

### RNA extraction and RT-PCR analysis

Total RNA was extracted from 1 × 10^7^ excysted oocysts using the RNeasy mini purification kit (Qiagen Gmbh, Hilden, Germany), following the manufacturer’s protocol for yeast. The preparation was digested with Dnase RQ1 (Promega Corporation, Madison, WI, USA) for 1 h at 37 °C and purified using the RNeasy mini purification kit (Qiagen Gmbh, Hilden, Germany), following the RNA clean up protocol. The reverse transcriptase (RT) reaction was performed on 3 μg of total RNA using the primers specified in **Supplementary Table 1**. The PCR amplification consisted of 30 cycles (60 s at 94°C, 90 s at 55 °C, and 60 s at 72 °C), followed by an extension cycle (10 min at 72 °C) on a GenAmp PCR System 2400 (Perkin-Elmer, Norwolk, CT, USA). The positive control reaction was performed, as above, on 3 μg of total RNA with primers for the *Cpa135* (Tosini et al., 2004). In negative controls, RT reactions were omitted.

### Expression of recombinant rhomboid in *E. coli* strains and preparation of antisera

A strategy for cloning the recombinant 6His-CpRom1 has been reported in Bertuccini et al. (2024). Similarly, 6His-CpRom2 and 6His-CpRom3 were cloned in pQE80 expression vector (Qiagen GmbH, Hilden, Germany) using extended primers with opportune restriction sites at their ends (*Sac*I and *Sma*I for 6His-CpRom2, and *Bam*HI and *Sma*I for 6His-CpRom3). The primer sequences are reported in **Supplementary Table 1**. Both amplifications were performed with 2X Phusion Flash High-fidelity PCR Master Mix (Finnzymes) using 80 ng of cDNA (see above) as template for each reaction as follows: 95 °C for 5 min, 35 cycles of 94 °C for 15 s, 50 °C for 15 s, 72 °C for 4 min, and a final extension at 72 °C for 10 min in a Veriti 96-well thermal cycler (Applied Biosystem). Amplicons were purified with a QIAquick PCR Purification Kit (Qiagen GmbH, Hilden, Germany) and digested with *Sac*I plus *Sma*I restriction enzymes (New England Biolabs) for 6His-CpRom2 and *Bam*HI and *Sma*I for 6His-CpRom2 and 6His-CpRom3, respectively. Resulting fragments were then ligated in the *Sac*I plus *Sma*I or *Bam*HI plus *Sma*I digested pQE80 vector (Qiagen GmbH, Hilden, Germany) for cloning 6His-CpRom2 or 6His-CpRom3, respectively, with the Quick Ligase kit (New England Biolabs) and used to transform the *Escherichia coli* M15 strain. Positive clones were selected on LB agar plates with 100 µg/mL ampicillin and 25 µg/mL kanamycin by PCR screening, and proper fusions with histidine tag were checked by sequencing. Purification of 6His-CpRom2 and 6His-CpRom3 was as follows: overnight cultures of recombinant bacteria were inoculated in 1 L of LB with 100 µg/mL ampicillin and 25 µg/mL kanamycin and cultured at 37 °C until a 0.6–0.8 OD was reached; then, 1 mM IPTG was added and cultured for an additional 3 h. Bacteria pellets were obtained by centrifugation at 1,500 × *g* for 10 min, then resuspended in 100 mL of ice-cold PBS and centrifuged again at 3,300 × *g* for 20 min. The pellets were lysed in 20 mL of denaturing buffer A (100 mM NaH_2_PO_4_, 10 mM Tris-HCl, and 6 M guanidine-HCl, pH 8.0) and stirred overnight at 25 °C. The lysates were clarified by centrifugation at 9,400 × *g* for 30 min. Then, 5 mL of 50% Ni-NTA resin was added and gently mixed by stirring for 60 min at 25 °C; the slurries were packed in a 10-mL column and the resin was washed with 40 mL (8 × 5 mL wash) of buffer C (100 mM NaH_2_PO_4_, 10 mM Tris-HCl, and 8 M urea, pH 6.3). Purified proteins were eluted with 4 aliquots of 2.5 mL of buffer D (100 mM NaH_2_PO_4_, 10 mM Tris-HCl, and 8 M urea, pH 5.9) and finally with 4 aliquots of 2.5 mL of buffer E (100 mM NaH_2_PO_4_, 10 mM Tris-HCl, and 8 M urea, pH 4.5). Eluted fractions were pooled and extensively dialyzed against PBS with increasing concentrations of glycerol (from 10% to 50%) in a Slide-A-Lizer™ (Thermo Fisher) dialysis cassette with a 10-kDa cutoff.

### Western blot, mouse sera preparation, and dot blot analysis

Solubilization of oocyst/sporozoite proteins was obtained in 20 μL of lysis buffer [10 mM Tris-HCl, pH 7.5, 10 mM DDT, 1 mM EDTA, 1% SDS, 1% Triton X-100, 0.5% sodium deoxycholate, plus Protease Inhibitor Cocktail (Sigma Aldrich) added immediately prior the use], incubated at room temperature for 5 min in a thermomixer, and mixed at 1,000 rpm (Eppendorf) at 70°C for 5 min. Before loading in sodium dodecyl sulfate–polyacrylamide gel electrophoresis (SDS-PAGE), 20 μL of Laemmli buffer was added to the samples and incubated at 95 °C for 5 min. Electrophoresis was performed on a MiniProtean apparatus (Bio-Rad) with precast gels 4%–15% (Bio-Rad). Expression of recombinant proteins was checked on 3 mL of bacterial culture induced for 3 h with 1 mM of IPTG, pelleted and lysed with Laemmli sample buffer, loaded and ran on 4%–20% TGX™ SDS-PAGE precast gels (Bio-Rad, Hercules, CA, USA), and finally tested in Western blot as below. Aliquots of 20–50 μL of purified recombinant proteins were incubated at 95 °C for 5 min in Laemmli sample buffer before loading on SDS-PAGE as above. Gels were transferred to nitrocellulose membranes (Bio-Rad), which were then blocked in TNT buffer (0.1 M Tris-Cl, pH 7.5, 150 mM NaCl, and 0.1% Tween-20) with 5% non-fat milk. Blots were incubated with primary antibodies for 1 h at room temperature. For recombinant 6His-CpRom1, 6His-CpRom2, and 6His-CpRom3, blots were incubated with mouse monoclonal RGS-His antibody (Qiagen) diluted 1:1,000. Blots for CpRom1 were probed as described in Bertuccini et al. (2024). Blots for native CpRom2 and CpRom3 were probed with mouse pre-immune and immune sera diluted 1:250. Three washing steps of 5 min each were conducted with TNT buffer plus 0.1% bovine serum albumin (BSA) before the incubation with secondary antibodies. For all blots, incubation with secondary antibodies was conducted for 1 h, at 25 °C, with goat anti-mouse immunoglobulin G (IgG)–horseradish peroxidase (HRP) conjugate (Bio-Rad, Hercules, CA USA) diluted 1:3,000. Three washing steps as above were conducted after incubation with secondary antibodies. Detection of proteins was performed using Pierce ECL substrate (Thermo Fisher), and bands were visualized with a ChemiDoc MP Imaging System (Bio-Rad, Hercules, CA, USA).

### Preparation of antisera for CpRom2 and CpRom3

Three Balb-C mice for each recombinant protein were immunized with 100 µg of protein plus complete Freund adjuvant as first inoculum, then after 30 days, with 100 µg of protein plus incomplete Freund adjuvant as second inoculum, and, after another 30 days, with 100 µg of protein in PBS. Finally, mice were bled 15 days after the last inoculum and 150–200 µL of serum was obtained from each mouse. Mice sera were tested by dot blot spotting approximately 100 ng of recombinant protein on nitrocellulose membrane in a Bio-Dot apparatus (Bio-Rad, USA). Membranes were treated and developed as for the Western blots (see above).

### Fractioning of E. coli extract in M15, Ruv3, and Ruv 5 *E. coli* strains

Vectors for expression of recombinant rhomboids were also used to transform *E. coli* strains RV-1337-3 (RuV3) and RV-1337-5 (RuV5) (Massey-Gendel et al., 2009). Each recombinant strain was inoculated in 8 mL of LB medium and left to grow at 37 °C up to 0.6 OD, then bacterial cultures were induced with 1 mM of IPTG and left to grow for an additional 3 h. Bacteria were pelleted by centrifugation at 2,040 × *g*, resuspended in 1 mL of H_2_O, and subjected to six cycles of 30 s of sonication at 75% strength in HD2070 Sonopuls (Bandelin Electronic, Berlin). Lysates were centrifuged at 10,000 × *g* for 15 min at 4 °C, pellets were resuspended in 50 μL of Laemmli buffer, and 10 μL was used for Western blot analysis. Supernatants were centrifuged at 90,000 × *g* in Optima-MAX TL-ultracentrifuge (Beckman-Coulter, USA) to collect membrane fraction, resuspended in 50 μL of Laemmli buffer, and 10 μL was used for Western blot analysis; 50 μL of ultracentrifuge supernatants was diluted with 4 × Laemmli buffer and 12.5 μL was loaded in SDS-PAGE as soluble protein fraction. Results are reported in **Supplementary Figure 1** (**Supplementary Data**).

### Indirect immunofluorescence assay

Oocysts and sporozoites were fixed on microscopic slides with 4% formaldehyde in PBS for 10 min, washed, and blocked with 2% BSA in PBS for 1 h. Sporozoites were permeabilized with PBS plus 0.1% Triton X-100 (Sigma-Aldrich) for 10 min at room temperature and then removed by washing the slides with PBS. In one experiment, permeabilization was omitted. The anti-rhomboid mouse sera were diluted from 1:100 to 1:1,000 in PBS plus 2% BSA and incubated for 1 h at room temperature. The anti-ID2 rabbit serum was used 1:1,000 in PBS plus 2% BSA and incubated for 1 h at room temperature in combination with anti-CpRom1 (mouse serum) 1:1,000. After washing in PBS, the slides were incubated for 1 h with goat anti-mouse antibody conjugated with fluorescein (FITC) (Bio-Rad, Hercules, CA), diluted 1:1,000 in PBS plus 2% BSA and with goat anti-rabbit antibody conjugated with rhodamine 1:1,000 (Bio-Rad, Hercules, CA). All incubations were performed at room temperature. Finally, nuclei staining was obtained with 300 nM DAPI (4′,6-diamidino-2-phenylindole) (Thermo Fisher) for 1 min at room temperature. Slides were then washed with PBS, and mounting medium (Thermo Fisher) was added before sealing the slides with the cover slides.

Staining procedures for HCT8-infected cells were conducted on a fixed micro-slides chamber (Thermo Fisher) probed with mouse primary antibodies for rhomboids and rabbit anti-sporozoite serum 1:1,000 (Trasarti et al., 2007). Mouse anti-rabbit Alexa (388)-conjugated antibody and goat anti-mouse Alexa (594)-conjugated antibody (Thermo Fisher) were used as secondary antibodies. Microslides were visualized with a fluorescence microscope (Zeiss-Axioplan 2), and images were elaborated with Axiovision 4.8.1 (Zeiss, Germany).

### *In vitro* infection on HCT-8 cell culture

Human colorectal adenocarcinoma cells (HCT-8) were maintained in RPMI 1640 medium (GIBCO/Invitrogen, Paisley, United Kingdom), enriched with 5% fetal bovine serum (FBS; GIBCO), 200 mM L-glutamine (Sigma, St. Louis, MO), 1% sodium pyruvate (Sigma), and 5% penicillin and streptomycin. These cells were cultured in flasks and kept at 37 °C in a humidified atmosphere with 5% CO_2_ and 85% humidity. Aliquots of 1 × 10^7^ oocysts were excysted and, after 30 min, were used to infect semi-confluent HCT8 cells. Multiplicity of infection was 10 oocysts for cell, and cultures were continued for 48 h on chamber slides.

### Ultrastructural analysis and immunolocalization of CpRom1

For immunolocalization of Cprom1, samples containing free sporozoites were fixed overnight at 4 °C with 4% paraformaldehyde and 0.1% glutaraldehyde in 0.1 M Na cacodylate buffer. Next, samples were rinsed in 0.1 M Na cacodylate buffer, dehydrated in ethanol serial dilutions, and embedded in LR White, medium-grade acrylic resin (London Resin Company, UK). Samples were polymerized in a 55 °C oven for 48 h, and ultrathin sections (90 nm) were collected on gold grids. The grids were floated on drops of PBS containing 0.1 M glycine for 10 min, washed with PBS, blocked with 5% normal goat serum/1% BSA in PBS for 30 min, and incubated overnight at 4 °C with the anti-ID2 (1:10) in PBS/0.1% BSA. After washing, the grids were incubated for 1 h with 10 nm gold-conjugated goat anti-mouse IgG (Sigma Aldrich) diluted 1:20, rinsed in PBS/0.1% BSA, followed by distilled water and air dried. Finally, samples were stained successively with 2% uranyl acetate and Reynolds lead citrate solution, and observed with a Philips EM208S transmission electron microscope (FEI - Thermo Fisher).

### Bioinformatics tools

General similarity searches were conducted on non-redundant GenBank databases using the BLAST program, available at https://blast.ncbi.nlm.nih.gov/Blast.cgi.

To identify protein coding sequences, ORFfinder at https://www.ncbi.nlm.nih.gov/orffinder/ and GenScan at https://pbil.univ-lyon1.fr/members/duret/cours/INSA/exercise4/pgscan.html were used. Searches for homologous proteins were performed with BlastP algorithm at:

http://cryptodb.org/cryptodb/ for *Cryptosporidium* spp. and *Gregarina niphandrodes*;
http://toxodb.org/toxo/ for *T. gondii*; and
http://plasmodb.org/plasmo/ for *P. falciparum*.

The evolutionary history was inferred using the Neighbor-Joining method, and bootstrap test was performed (500 replicates). The evolutionary distances were computed using the Poisson correction method and reported in the units of the number of amino acid substitutions per site. The analytical procedure encompassed 29 amino acid sequences. The pairwise deletion option was applied to all ambiguous positions for each sequence pair, resulting in a final dataset comprising 127 positions. Evolutionary analyses were conducted in MEGA12 (Kumar et al., 2024) utilizing up to four parallel computing threads. Prediction of TM domains was performed with TMHMM v.2 at https://services.healthtech.dtu.dk/services/TMHMM-2.0/ and at http://smart.embl-heidelberg.de or with DeepTMHMM-1.0 at https://services.healthtech.dtu.dk/services/DeepTMHMM-1.0/ (Hallgren et al., 2022). Newly predicted TMs were verified with AlphaFold (Fleming et al., 2025) at https://alphafold.ebi.ac.uk.

Clustal alignments were performed at http://www.ebi.ac.uk/Tools/msa/clustalo/. Bidimensional representations of CpRoms were obtained with Protter at http://wlab.ethz.ch/protter/start/ (Omasits et al., 2014), applying custom topology for the TM domains. Putative targets of rhomboids were identified by similarity searches at CryptoDB with the following proteins of *T. gondii*: TgMic2 (TGME49_201780), TgMic6 (TGME49_218520), TgMic12 (TGME49_267680), TgMic8 (TGME49_245490), and the *P. falciparum* thrombospondin-related anonymous protein (TRAP) (PF3D7_1335900.1) as queries in the BLAST program. Searches for protein–protein interaction were carried out in String database at https://string-db.org/cgi/input?sessionId=bV4mVyVVojwM&input_page_show_search=on.

## Results

### Identification of *C. parvum* rhomboids

**Figure 1:**
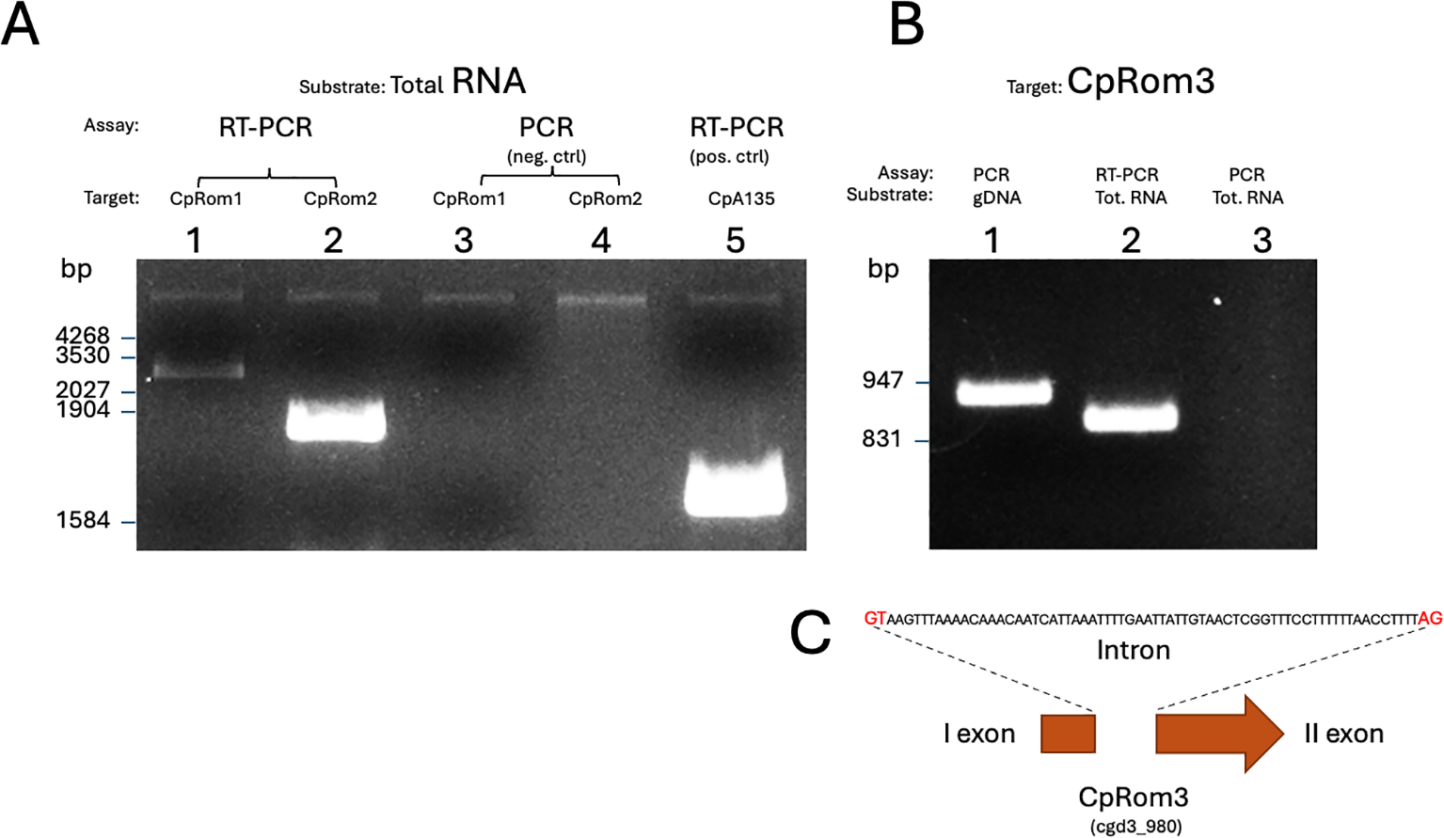
Expression of *C. parvum* rhomboids in sporozoites. **(A)** RT-PCR on sporozoite total RNA: lanes 1 and 2, reverse-transcribed mRNA for CpRom1 and CpRom2, respectively; lanes 3 and 4, PCR as in the previous lanes without RT reaction as negative control; lane 5, RT-PCR amplification for CpA135 as positive control (Tosini et al., 2004). **(B)** Lane 1, PCR amplification for CpRom3 on genomic DNA; lane 2, RT-PCR on total RNA for CpRom3; lane 3, PCR as in the previous lanes without RT reaction as negative control. **(C)** Scheme showing the two exons encoding CpRom3 and the interposed intron sequence.

CpRom1 identification was previously reported (Trasarti et al., 2007). In this study, two other predicted rhomboids were identified by a homology search conducted on the translated *C. parvum* (Iowa strain) genome, using various apicomplexan rhomboid sequences (i.e., known rhomboids from *T. gondii* and *P. falciparum*) as queries. Altogether, this parasite encodes for three functional rhomboids, and the assigned name, Crypto ID, and UniProt ID are reported in **Table 1**. We noted that there was a clear case of homonymy regarding the CpRom1 name, given that this name was assigned to three different proteins as reported in **Table 1** (Li et al., 2016; Castellanos-Gonzalez et al., 2019; Gao et al., 2021; Bertuccini et al., 2024). Therefore, the need for an unambiguous classification has prompted us to propose a revised nomenclature based on protein length, in decreasing order as reported in **Table 1**. Hence, these proteins were named CpRom1 (990 aa), CpRom2 (464 aa), and CpRom3 (282 aa), and such nomenclature will be used throughout the manuscript. Genes encoding CpRom1 and CpRom2, namely, cgd6_760 and cgd7_3020, respectively, are mono-exonic and do not contain introns. Differently, CpRom3, encoded by cgd3_980, is composed of two exons (**Figure 1**).

**Figure 2:**
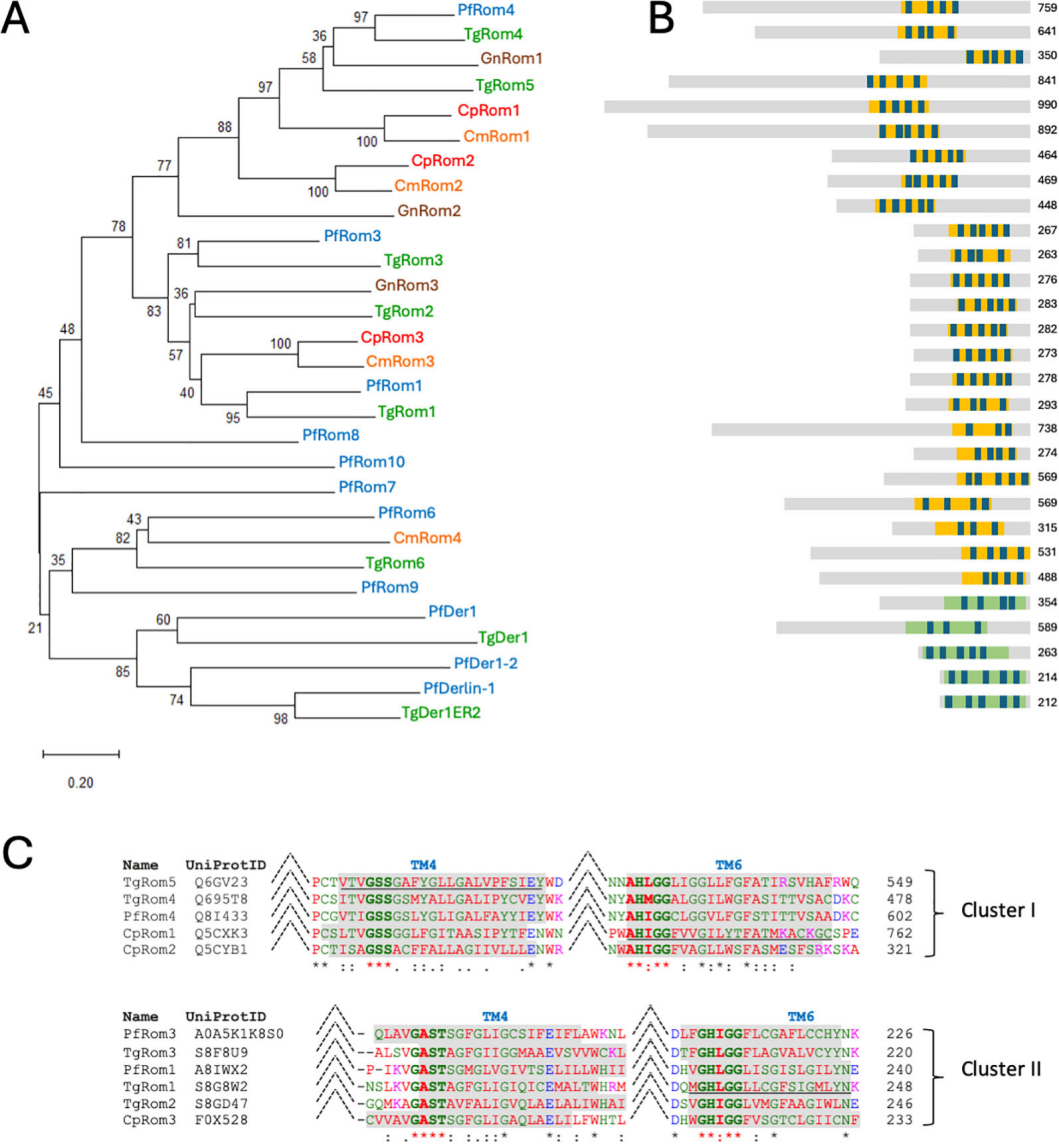
Evolutionary relationships of Apicomplexa rhomboids. **(A)** Neighbor-joining phylogenetic tree of the Apicomplexa rhomboids and rhomboid-related proteins (Derlins) is shown. The percentage of replicate trees in which the associated taxa clustered together in the bootstrap test is shown next to the branches. The tree is drawn to scale, with branch lengths in the same units as those of the evolutionary distances used to infer the phylogenetic tree. Protein names are colored according to species. **(B)** A schematic representation of proteins is proposed; the rhomboid domain is reported as yellow bars, while the Derlin domain is denoted as green bars. Transmembrane helices are indicated by blue segments. Transmembrane helices are indicated by blue segments (a list of TMs with their position is reported in **Supplementary Table 2**). The lengths of the proteins are indicated on the right. **(C)** ClustalO alignments with homologs of CpRom1, CpRom2, and CpRom3, which include the catalytic sites composed of TM4 and TM6 (see the text). Apicomplexan rhomboids can be classified in two distinct clusters by reason of the amino acids composing the catalytic sites. Shaded amino acids indicate predicted TM domains. Bold letters correspond to the catalytic dyad and the surrounding conserved amino acids. Underlined TMs were predicted by similarity and verified by AlphaFold. Dashed lines indicate interruptions in sequences and extensive alignments are reported as **Supplementary Material** (**Supplementary Figure 2**).

**Table 1:** List of the *C. parvum* rhomboids in this study.

Assigned name (CryptoDB ID/name)	UniProt ID	Amino acids	Chromosome localization	GO terms	Localization in sporozoites^1^	OOC/SPO^2^	Intracell. stages^2^	Proteomics	Alternative name with reference
*CpRom1 cgd6_760* *Peptidase S54 rhomboid*	Q5CXK3	990	6	Proteolysis Integral component of membrane Serine-type endopeptidase activity	Inner membrane complex or plasma membrane	Low	High	Oocyst wall proteome^3^ Sporozoite proteome^4^	ID2/CpRom (Trasarti et al., 2007). CpRom1 (Bertuccini et al., 2024)
*CpRom2* *cgd7_3020* *Rhomboid-like protein*	Q5CYB1	464	7	Proteolysis Integral component of membrane Serine-type endopeptidase activity	Unassigned	High	Low	No data	CpRom1 (Castellanos-Gonzalez et al., 2019)
*CpRom3* *cgd3_980* *Peptidase S54 rhomboid domain-containing protein*	F0X528	282	3	Proteolysis Integral component of membrane Serine-type endopeptidase activity	Unassigned	Medium	High	Oocyst wall proteome^5^	CpRom1 (Li et al., 2016). ^6^CpRom1 (Gao et al., 2021)

^1^Localization were based on results reported in Guérin et al. (2023).

^2^Espression values were based on Mirhashemi et al. (2018).

^3^Proteomics data based on Truong and Ferrari (2006).

^4^Proteomics data based on Sanderson et al. (2008).

^5^Wang et al. (2022).

^6^The discovery of this rhomboid has been presented as novel and independent from that of Li et al. (2016).

### Expression analysis of CpRom genes in sporozoites

To ascertain the expression of the three CpRom genes, three couples of primers were designed based on their genomic sequences and used for RT-PCR experiments on total RNA extracted from excysted sporozoites. As shown in **Figure 1**, all these genes are expressed in the sporozoites (**Figure 1A**: lanes 1 and 2 and **Figure 1B**: lane 2). PCR amplifications without previous RT were performed on total RNA as negative controls of the experiment (**Figure 1A**: lanes 3 and 4 and **Figure 1B**: lane 3) to exclude the presence of genomic DNA in total RNA samples. The larger size of the amplicon for CpRom3 on genomic DNA (**Figure 1B**: lane 1) with respect to that on retro-transcribed RNA (**Figure 1B**: lane 2) confirmed that this gene contained an intron. The genomic CpRom3 amplicon (**Figure 1B**: lane 2) was sequenced to confirm the intron sequence. A schematic representation of the intron and its sequence is reported in **Figure 1C**.

### Phylogenetic comparison of *C. parvum* rhomboids with other apicomplexan rhomboids

The *C. parvum* rhomboids identified were then compared with predicted proteins of other apicomplexan parasites in a genome-wide search to produce a representative phylogenetic tree of Apicomplexa rhomboids (**Figure 2A**). This analysis was conducted on the genomes of reference strains fully sequenced and annotated in EuPathDB, and *Cryptosporidium muris*, which is also annotated in CryptoDB, was also included as a representative of the *Cryptosporidium* species infecting the stomach instead of the intestine. The phylogenetic tree in **Figure 2A** shows that CpRom1 and CpRom2 (in red in the tree) are strictly related to PfRom4, TgRom4, and TgRom5, involved in cleaving the micronemal adhesins (Brossier et al., 2005; Baker et al., 2006; Rugarabamu et al., 2015). Differently, CpRom3 (also in red in the tree) appears to be related to a different cluster that includes PfRom3 and TgRom3. Genomic comparison also showed that none of the *C. parvum* rhomboids could be assigned to the PARL cluster. **Figure 2B** shows a schematic representation of these apicomplexan rhomboids with their lengths and distribution of the TM domains composing the rhomboid domain.

Of note, the strictly related *C*. *muris* showed an additional fourth rhomboid that we named CmRom4 and that clustered with the PARL-like rhomboids (see also below).

### Identification of structural domains and catalytic sites of *C. parvum* rhomboids

To identify structural domains and the catalytic sites along the amino acid sequences of the three *C. parvum* rhomboids, their sequences were aligned with those of the most similar rhomboids from *P. falciparum* and *T. gondii*, which are the most characterized among the apicomplexan rhomboids. These alignments led to the identification of further TM domains that were not previously identified and were confirmed by AlphaFold modeling. Novel TM domains were revealed in TgRom1, TgRom4, TgRom5, PfRom4, and CpRom1 and are reported in **Supplementary Figure 2**. In **Figures 2C**, the extrapolated alignments of the TM4 and TM6 that compose the catalytic sites of these rhomboids are shown. As expected, CpRom1 and CpRom2 clustered with TgRom4, TgRom5, and PfRom4 (Cluster I), all sharing the following conserved consensuses: a GSS motif at the fourth TM and an AHXGG motif at the sixth TM (**Figure 2C**). Differently, CpRom3 belonged to a separate lineage, which included PfRom3, TgRom3, PfRom1, TgRom1, and TgRom2 (Cluster II). This group was characterized by the following conserved consensuses: a GAST motif at the fourth TM and a GHIGG consensus at the sixth TM (**Figure 2C**). Bidimensional representations of the three *C. parvum* rhomboids with the dislocation of the TM domains and the catalytic sites in relation to the cell membrane are reported in **Supplementary Figure 3**. Finally, these results showed that the active apicomplexan rhomboids, except for PARLs, are composed of seven TM domains and can be classified as mixed secretase (Lemberg and Freeman, 2007). Furthermore, these apicomplexan rhomboids can be sub-grouped into two different clusters based on the conserved residues around the catalytic dyad.

### PARL rhomboids in *Cryptosporidium* genus

A survey to identify rhomboids in genomes of other *Cryptosporidium* species revealed that species affecting the intestine (i.e., *C. parvum*, *C. hominis*, and *Cryptosporidium meleagridis*) showed a similar genomic content with only three rhomboids (data not shown); differently, two species, namely, *C. muris* and *Cryptosporidium andersoni*, which infect the stomach, encode for four rhomboids. None of the *C. parvum* rhomboids belong to the PARL group of mitochondrial rhomboids, and this is not surprising given that this parasite, as well as other *Cryptosporidium* species, lacks a functional mitochondrion. However, the similarity search for rhomboids in the Apicomplexa genus revealed that both *C. muris* and *C. andersoni* possess PARL-like genes. The protein CmRom4, the PARL-like rhomboid of *C. muris*, was more like the PARL proteins of *P. falciparum* and *T. gondii* (see **Figure 2A**) than the other *C. parvum* rhomboids. A bidimensional representation of CmRom4 PARL-like rhomboid is reported in **Supplementary Figure 4** and, differently from the *C. parvum* rhomboids, shows only six TM domains.

**Figure 3:**
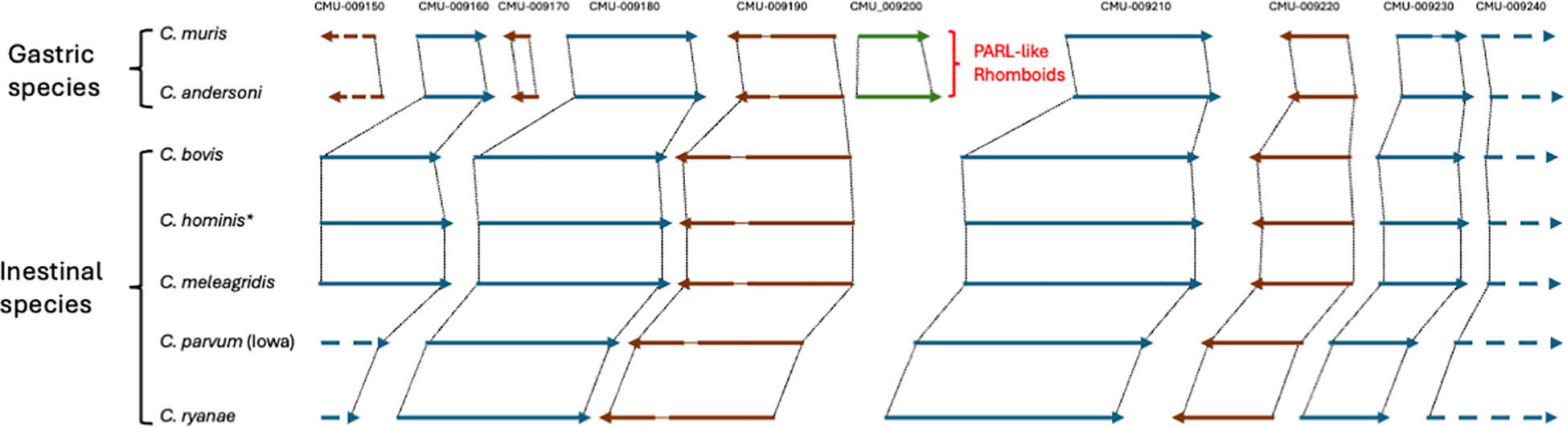
Schematic representation of the genomic regions that surround the PARL-like rhomboid genes in *C. muris* and in *C. andersoni* in comparison with the corresponding regions in other *Cryptosporidium* species. PARL-like rhomboids are present in “gastric species” whereas they are lacking in “intestinal species”. The reconstruction was based on the synteny obtained from CryptoDB for the CMU_009200 gene (CmRom4). The IDs at the top correspond to the *C. muris* genes, and the PARL-like rhomboid genes are represented in green. Arrows indicate the genes and the transcription direction (blue arrows from left to the right and brown arrows from right to the left). The thin lines inside the arrows indicate introns interrupting the coding sequences. Dotted lines connect orthologs through the different species. Dashed arrows represent interrupted genes. *There are four different annotated genomes for *C. hominis* in CrytpoDB, which are almost identical in this region and are synthesized in a unique scheme.

Overall, we found out that species that infect the intestine, like *C. parvum*, *C. hominis*, and *C. bovis*, lack a PARL-like gene. By contrast, *Cryptosporidium* species infecting the stomach, namely, *C. muris* and *C. andersoni*, possess a PARL-like rhomboid. There is no evidence for the presence of a mitochondrion in *C. andersoni*, although this species possesses some genes involved in the aerobic metabolism that are absent in *C. parvum* and *C. hominis* (Liu et al., 2016). Importantly, a mitochondrion has been identified at the ultrastructural level in *C. muris* (Uni et al., 1987). The genomic regions surrounding the PARL-like genes in *Cryptosporidium* species, by comparison, revealed a precise removal of these genes in species without mitochondrion, like *C. parvum*, *C. hominis*, *C. bovis*, and others (**Figure 3**). Remarkably, except for the precise excision of the PARL-like genes, the synteny, namely, the distribution of the other surrounding genes along the chromosome, is essentially conserved among these different species.

### Expression of recombinant *C. parvum* rhomboids in *Escherichia coli*

**Figure 4:**
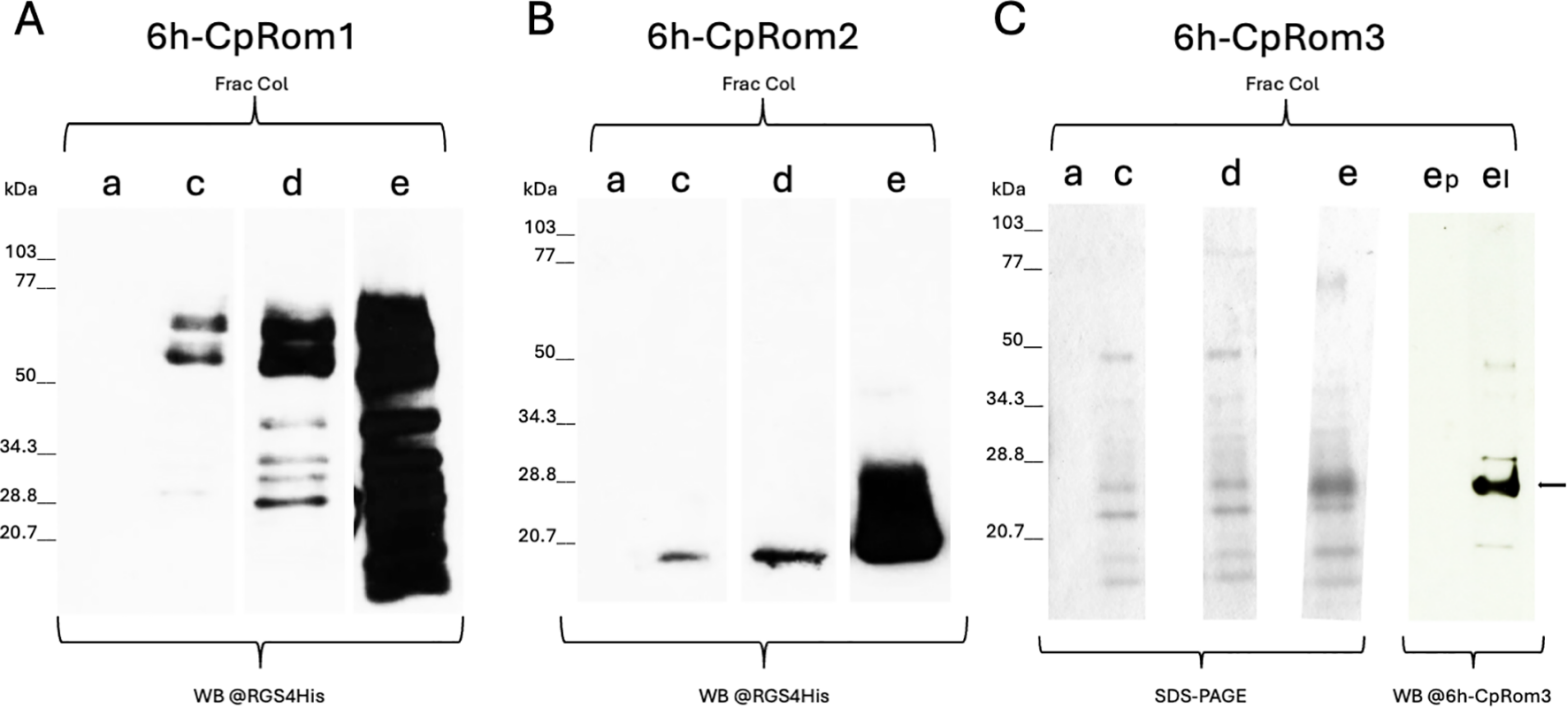
Purification on Ni-NTA-resin of recombinant rhomboids expressed in *Escherichia coli*. Fractions are marked at the top of the lanes with the corresponding buffers: a, binding buffer; c, wash buffer; d and e, elution buffers (see Material and Methods). **(A)** Western blot analysis of 6h-CpRom1 elution fractions probed with mouse anti-RGS4His monoclonal antibody (MoAb). **(B)** Western blot analysis of 6h-CpRom2 elution fractions probed with mouse anti-RGS4His MoAb. **(C)** SDS-PAGE of elution fractions 6h-CpRom3 and Western blot analysis of E fraction with preimmune (ep) and immune (ei) in-house mouse serum for 6h-CpRom3. Twenty microliters of eluate was loaded in each lane, and total protein concentration ranged between 10 and 100 ng.

Coding sequences for the three rhomboids were cloned in expression vectors to produce recombinant proteins fused with a histidine tag. Portions of the coding sequences avoiding the multi-spanning rhomboid domain were also cloned, so as to favor the expression of soluble portions of the proteins to obtain specific peptides for immunization. Surprisingly, only the full-length tagged proteins, but not their shorter fragments, were expressed by bacteria. Schematic representations of the whole set of constructs and their expression results are reported (**Supplementary Figure 4**). These tagged rhomboids were tracked by a specific monoclonal antibody (MoAb) that marks the N-terminal histidine tag of the recombinant forms (**Figure 4**). However, column fractions of 6h-CpRom3 were scarcely labeled by this specific MoAb, differently from the forms expressed in the *E. coli* Ruv5 (see below), and purification fractions of 6h-CpRom3 were checked by SDS-PAGE and Western blot with the specific in-house mouse serum (**Figure 4C**). Therefore, the full-length recombinant rhomboids produced were named 6h-CpRom1 (Bertuccini et al., 2024), 6h-CpRom2, and 6h-CpRom3, and their expected molecular masses were 110, 51, and 35 kDa, respectively. However, the expression in a conventional recipient *E. coli* strain for his-tagged constructs (M15 strain) gave fragmented and scarcely soluble products that required the presence of denaturing agents (i.e., guanidine and urea) for the purification of the recombinant peptides (**Figure 4**). Thus, the recombinant 6h-CpRom2 and 6h-CpRom3 were synthetized as unique peptides of 20 and 25 kDa, respectively. The cutting of these recombinant proteins was probably due to the action of bacterial proteases before lysis in denaturing conditions. The processing in bacteria was particularly evident in 6h-CpRom1 that was cleaved several times, as evidenced by the multiple bands observed in the Western blot (**Figures 4A**, lanes c–e). In any case, the shorter 6h-CpRom2 and 6h-CpRom3 were suitable for the chromatographic purification and the preparation of specific mouse antisera. The successful production of specific IgGs was checked by dot blots at 60 and 75 days post-immunization (**Supplementary Figure 5**).

To produce functional recombinant rhomboids localized in the cell membrane, the expression of these constructs, as well as that of 6h-CpRom1, was attempted in alternative *E. coli* strains that have been improved to produce functional membrane proteins (Massey-Gendel et al., 2009). Therefore, these strains were transformed with the 6h-CpRom1, 6h-CpRom2, and 6h-CpRom3 constructs and induced to express the recombinant rhomboids. The induced bacterial cultures were lysed, homogenized, and separated in subcellular fractions that were analyzed by Western blot (**Supplementary Figure 1**). None of the assayed recipient strains was compatible with the proper expression of 6h-CpRom1, and this recombinant protein was always accumulated in cytoplasmic inclusion bodies regardless of the strain. In **Supplementary Figure 1**, the impaired expression in Ruv3 is shown, and similar results were obtained with the other strains (data not shown). On the other hand, the expected bands of 51 and 35 kDa for 6h-CpRom2 and 6h-CpRom3, respectively, were identified in the membrane fraction of the Ruv5 strain. Therefore, the placement in a cell membrane was required to obtain the integral forms of these two proteins.

### Identification of *C. parvum* rhomboids in oocyst and sporozoite proteomes

**Figure 5:**
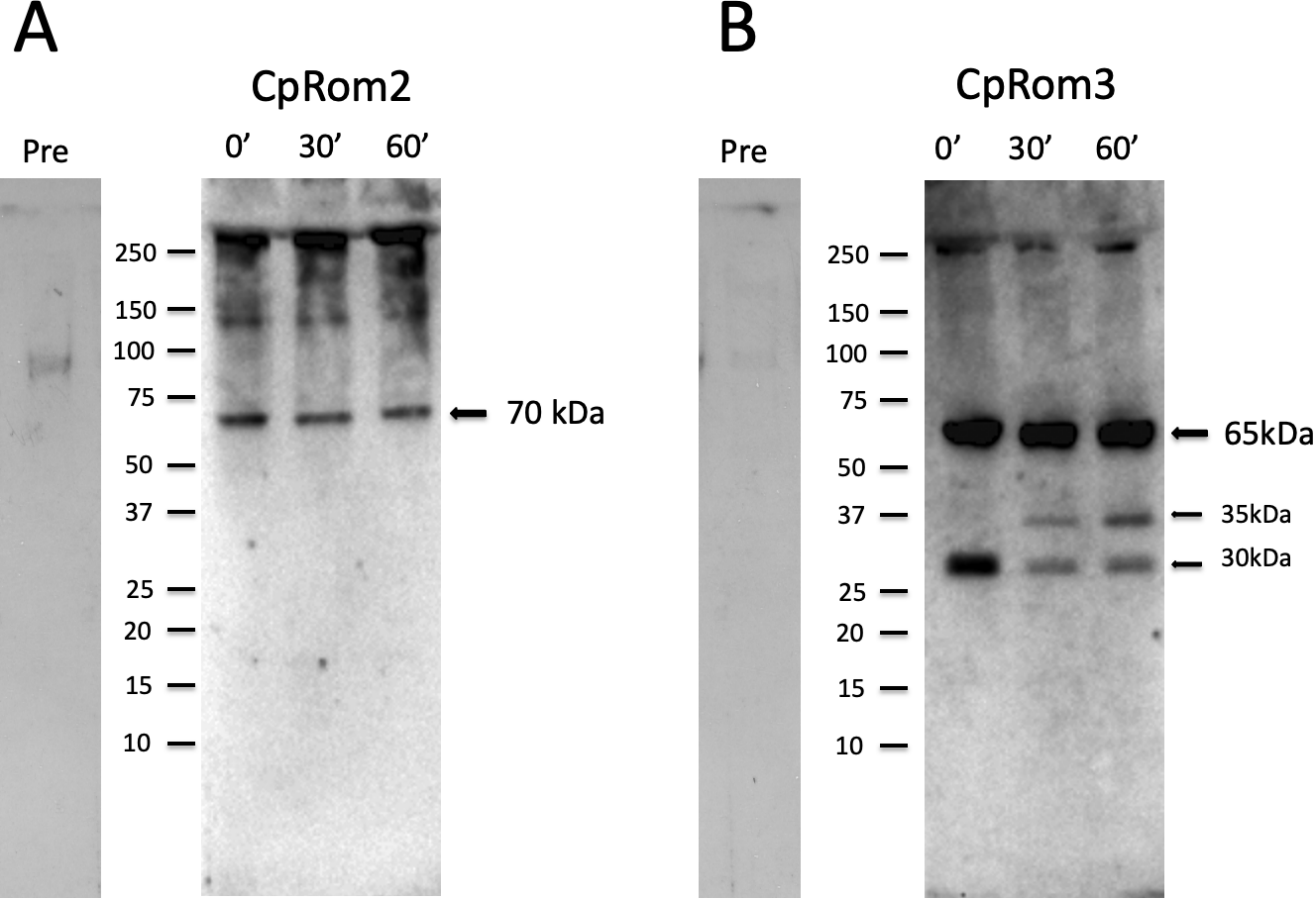
Western blot analysis of oocyst and sporozoite lysates at different times from the induction of excystation. **(A)** Lysates were probed with polyclonal mouse anti-serum for CpRom2 (dilution 1:500). **(B)** Lysates were probed with polyclonal mouse anti-serum for CpRom3 (dilution 1:100). Numerals at the top indicate minutes from the excystation start. Lanes marked with Pre show Western blots probed with pre-immunized mouse sera respectively for CpRom2 (left) and for CpRom3 (right). Molecular standards are reported on the left side of the blots. Approximately 20 μg of total proteins was loaded in each lane.

To identify the native *C. parvum* rhomboids, whole sporozoite lysates were analyzed by Western blot using positive sera (**Figure 5**). As observed for CpA135 (Tosini et al., 2004), the expression of certain proteins can change within the first hour after the excystation; therefore, we decided to assay rhomboids at three different times: 0, 30, and 60 min after the start of the excystation.

Using anti-CpRom2 serum, we observed a unique band of approximately 70 kDa of constant intensity (**Figure 5A**) from the oocyst (time 0) to the excysted sporozoites (time 30 and 60 min from the excystation start). The difference between the expected size of approximately 50 kDa and the observed size of 70 kDa can be the result of a post-translational modification such as the addition of glycosides or other covalently linked molecules. Differently, the native CpRom3 appeared as a triplet as revealed by the anti-CpRom3 serum: one of approximately 70 kDa and two smaller bands of approximately 30 and 35 kDa that vary in intensity from the beginning up to 1 h from the excystation start (**Figure 5/B**). The 35-kDa band corresponded exactly to the expected size of CpRom3, which appeared only after the excystation, and its amount slightly increased in 1 h after the excystation (**Figure 5B**). In contrast, the 30-kDa form decreased during the excystation (**Figure 5B**). The presence of three forms of this protein can be explained by processes that modify CpRom3 in the sporozoite cell. Some rhomboids, by paradox, can assume a dimeric form in SDS-PAGE (Kreutzberger and Urban, 2018), and this may explain the 70-kDa form of CpRom3, which is exactly double the value of the expected 35 kDa. In both panels, larger extra bands can be observed, and insoluble residues of both rhomboids that did not migrate in the gel matrix can be observed at the bottom of each well (bands over 250 kDa). In **Figures 5A**, a smearing of CpRom2 can be observed, which reaches up to form a band of between 100 and 150 kDa. The insoluble forms, as well as the smear in CpRom2 lanes, could be explained with strong aggregation of rhomboids with residues of membranous structures. It should be mentioned that we used a strong solubilization procedure for rhomboids (see Material and Methods) to reduce the clumping observed (data not shown) with conventional denaturing treatment (i.e., incubation with Laemmli buffer).

Overall, the three *C. parvum* rhomboids, including the CpRom1 previously described (Bertuccini et al., 2024), are present in the oocyst and expressed in the excysted sporozoites.

### Different localization of *C. parvum* rhomboids in sporozoite

The specific sera for *C. parvum* rhomboids were also used to localize these proteins, and the colocalization with the nucleus marked with DAPI was used for placing the rhomboids with respect to the anteroposterior axis of sporozoites.

**Figure 6:**
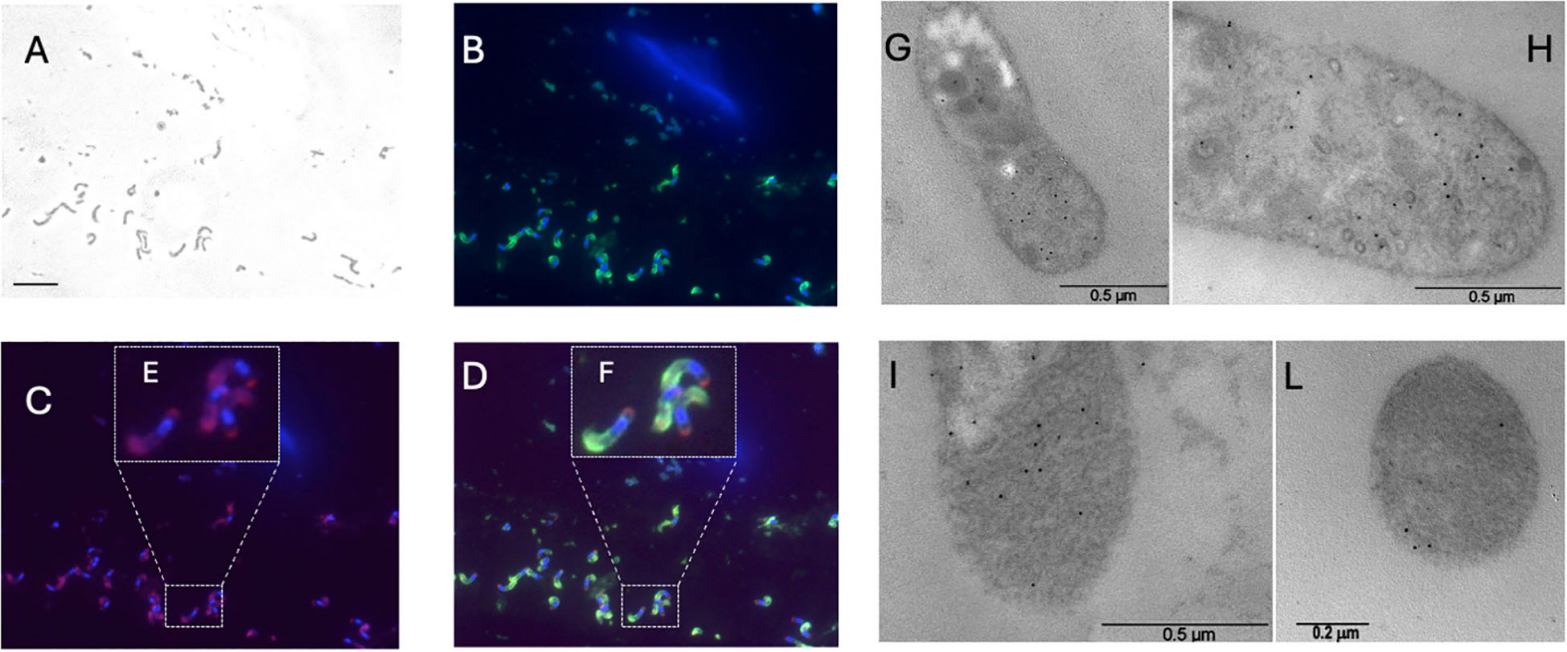
Localization of CpRom1 in excysted sporozoites by mouse anti-CpRom1 (IFA in **A–D**) and by rabbit anti-ID2/Cprom1 (immune-electron microphotographs in G–L). **(A)** Excysted sporozoites in white field. **(B)** Sporozoites labeled with DAPI (blue) and anti-sporozoite serum (green). **(C)** Anti-CpRom1 serum. **(D)** Merge of B and D. **(E, F)** Enlarged portions of C and D, respectively (dotted rectangles), to highlight labeling at the anterior and posterior pole of sporozoites. Scale bar in A corresponds to 10 μm. **(G, H)** Immunoelectron microscopy of *Cryptosporidium* sporozoite ultrathin sections at level of the apical pole, immune-gold particle-labeled area around the micronemes. **(I–L)** Ultrathin sections at the level of the crystalloid body (posterior pole): longitudinal section **(I)** and cross-section **(L)**; immuno-gold particles are distributed in this area.

For CpRom1, we also used a rabbit antiserum for sporozoite antigens that intensely stained the apical surface of sporozoites and the parasitophore vacuole (Tosini et al., 2019). Therefore, the combination of nucleus (blue) and the apical surface (green) labeling revealed that the CpRom1 had a dual location: one apical, evenly distributed, and, apparently, inside the cell membrane; and a second accumulation point that was instead exactly at the posterior pole of the sporozoites (**Figures 6A–D**). The immune electron-microscopy experiments confirmed the dual distribution of CpRom1 at the apical and posterior poles of sporozoites, as well as its internal localization at the apical pole (**Figures 6G–L**).

**Figure 7:**
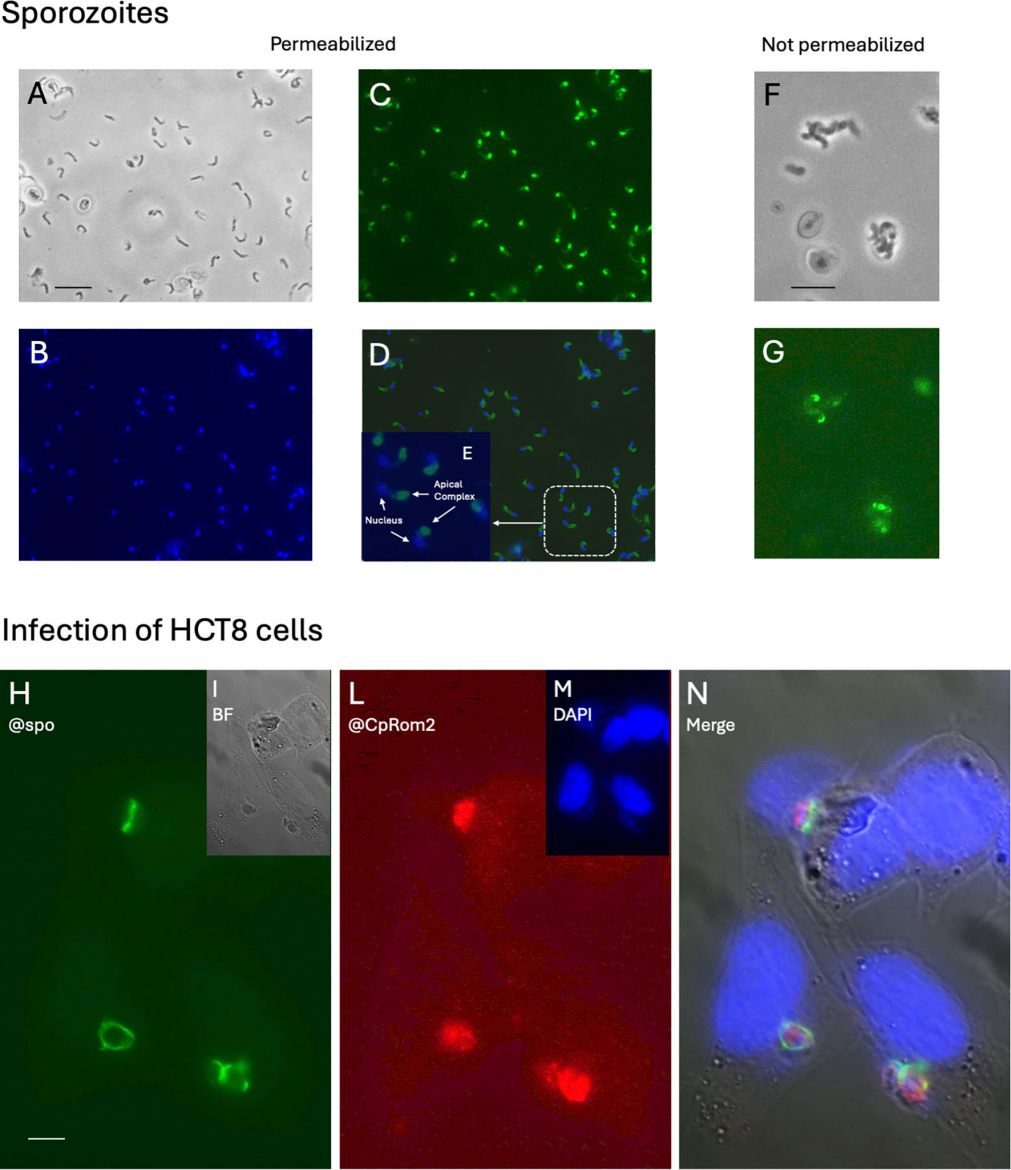
Immunolocalization of CpRom2 in excysted sporozoites and in infected HCT8 cells. **(A–E)** Colocalization on permeabilized excysted sporozoites. **(A)** Excysted sporozoites in white field. **(B)** Sporozoites labeled with DAPI (blue) and anti-sporozoite serum (green). **(C)** Anti-CpRom2 serum. **(D)** Merge of B and C. **(E)** Digital magnification of white dotted rectangle in D. **(F, G)** Immunolocalization of CpRom2 on non-permeabilized excysted sporozoites. **(F)** Excysted sporozoites in white field. **(G)** Immunofluorescence on the same field with anti-CpRom2 serum. **(H–N)** Colocalization of CpRom2 in infected HCT8 cells at 48 h PI. **(H)** Parasitophorous vacuoles at different stages labeled with antisporozoite rabbit serum and mouse anti-rabbit Alexa (388)-conjugated antibody. **(I)** Microscopic field observed with white light (BF = bright field). **(L)** Parasitophorous vacuoles labeled with mouse anti-CpRom2 and goat anti-mouse Alexa (594)-conjugated antibody. **(M)** DAPI staining of the nuclei. **(N)** Digital merging of H, I, L, and M images. Scale bar in H corresponds to 10 μm.

CpRom2 was instead distributed exclusively in the anterior portion of the sporozoite around the apical complex (**Figure 7**). This localization was evident in the digital magnification of few sporozoites in **Figure 7E**. We also tried to place this rhomboid in relation to the external membrane repeating this immunolocalization on non-permeabilized sporozoites, and, as shown in **Figures 7F, G**, only some sporozoites (i.e., approximately two out of six sporozoites within 30 min from the excystation start in this experiment) could be labeled in the upper part of the apical end. This result suggested that this rhomboid is accumulated in some apical organelle, probably the micronemes, and progressively transported to the apex of sporozoite.

**Figure 8:**
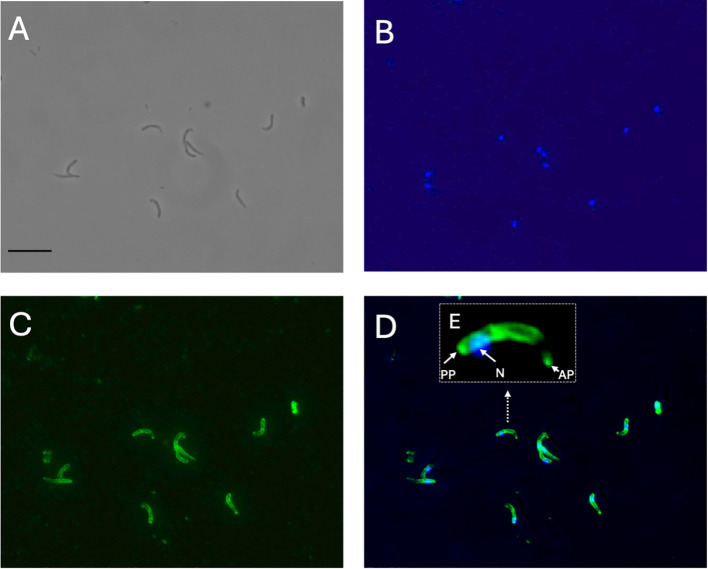
Localization of CpRom3 in excysted sporozoites (1,000 ×). **(A)** Excysted sporozoites in white field. **(B)** Sporozoites’ nuclei labeled with DAPI (blue). **(C)** Anti-CpRom3 serum. **(D)** Merge of B and C images. **(E)** Digital magnification of the sporozoite in the white dotted rectangle in D to localize the distribution of fluorescence along the sporozoite. Scale bar in A corresponds to 10 μm.

Differently, CpRom3 was observed to be evenly distributed along the sporozoite surface (**Figure 8**), with a thickening exactly at the apical end of the sporozoite as shown with a digital magnification (**Figure 8E**).

Finally, these results clearly indicated that the three *C. parvum* rhomboids were coexisting in excysted sporozoites, although their spatial localization was different from the other along the sporozoite cell.

### CpRom2 is present in the parasitophore vacuole in the first stage of infection

To detect rhomboids in the intracellular stages of the parasite in the first 48 h post-infection, we also tested HCT8-infected cells by immunofluorescence. While we did not observe specific labeling for CpRom1 (**Supplementary Figure 6**) and CpRom3 (data not shown), CpRom2 was still present in the parasitophorous vacuoles (PVs) at different intracellular stages (**Figures 7H–N**). In fact, the rabbit antiserum labeled in brilliant green the structure of PV (Tosini et al., 2019) and the mouse antiserum for CpRom2 marked in red the parasites inside the PV.

### *In silico* identification of substrates for *C. parvum* rhomboids

The cutting sites of rhomboids are composed of a few amino acids in the TM domain of the substrate membrane proteins (Strisovsky et al., 2009). However, a similarity search using the short consensuses of various proven rhomboid substrates in *C. parvum* genome did not reveal any consistent homology among the membrane proteins of this parasite. Therefore, the search for the putative targets of the three *C. parvum* rhomboids was based on the following criteria: high similarity with other apicomplexan proven substrates; the presence of one or more well-recognizable TM domains; the co-expression with rhomboids in the sporozoite stage; and, possibly, the colocalization with a rhomboid in a subcellular structure.

The similarity search on the *C. parvum* genome used the following substrates of apicomplexan rhomboids (Zhou et al., 2004): TgMic2 (TGME49_201780), TgMic6 (TGME49_218520), TgMic12 (TGME49_267680), and TgMic8 (TGME49_245490) of *T. gondii* and the thrombospondin-related anonymous protein (PF3D7_1335900) of *P. falciparum.* These proteins share a micronemal localization and a modular architecture that implies repeated thrombospondin domains and/or EGF domains, which are involved in the progression toward and the attachment to the host cell, and the removal from the parasite surface mediated by a rhomboid cleavage. This first step revealed a total of 18 C*. parvum* proteins, which were very similar to two or more template proteins of *T. gondii* and *P. falciparum*. *C. parvum* proteins without an identifiable TM domain were eliminated from this group and only 10 proteins remained candidates as rhomboid substrates (**Table 2**).

**Table 2:** Putative targets of *C. parvum* rhomboids.

CryptoDB ID	UniProt ID length	Name (UniProt)	GO terms	Position of TM (aa)	MS/MS data (peptides in oocyst/sporozoite stage)	Localization in oocyst/sporozoite^1^	OOC/SPO^2^	Intracell stages^2^	Similarity in ToxoDB: name and *E* value
Identified by similarity search and String analysis									
cgd2_1590	Q5CTT8 488 aa	Extracellular protein with signal peptide, 5xEGF and apple domains	Proteolysis extracellular region Calcium ion binding Protein binding	13–29	None	Oocyst wall	High	Medium	Calcium-binding egf domain-containing protein CBDP2 (TGME49_318540); 4e-49
cgd6_670	Q5CXK9 1607 aa	Extracellular protein with a signal peptide, clostripain-like caspase/hemoglobinase domain, notch domain and 2 EGF domains	Integral component of membrane Calcium ion binding	1,528–1,550	Yes (insoluble excysted fraction^3^)	Oocyst wall	Low	Low	Calcium-binding egf containing protein CBDP3 (TGME49_269930); 2e-62
Identified only by similarity search									
cgd5_3420	Q5CRC0 3,869 aa	TRAP-C2 extracellular protein	Integral component of membrane	3,758–3,780	One (oocyst wall^4^)	Inner membrane complex or plasma membrane	Low	Low	Sushi domain (scr repeat) containing protein (TGME49_223480); 1e-176
cgd2_3080	Q5CTG7 391 aa	CpTSP10 protein	Integral component of membrane	364–386	None	Microneme	Low	High	Sushi domain (scr repeat) domain-containing protein (TGME49_223480); 6e-21
cgd5_4470	Q5CQ18 656 aa	CpTSP7 extracellular membrane associated protein with a signal peptide followed by 2 TSP1 repeats, an EGF domain and a transmembrane region	Integral component of membrane	563–582	None	Microneme	Low	High	Sushi domain (scr repeat) domain-containing protein (TGME49_223480); 2e-35
cgd6_780	Q5CXK1 625 aa	Putative extracellular protein	Integral component of membrane	Multispanning 17–36 599–621	None	Microneme	High	Low	Sushi domain (scr repeat) domain-containing protein (TGME49_223480); 1e-24
cgd6_800	Q5CXK0 457 aa	CpTSP9, extracellular protein with 3 TSP1 domains and an EGF domain	Integral component of membrane Calcium ion binding	397–416	None	Microneme	Low	High	Sushi domain (scr repeat) domain-containing protein (TGME49_223480); 1e-30
cgd1_3500	Q5CSA5 687 aa	Thrombospondin-related adhesive protein	Integral component of membrane	622–644	Many (ooocyst wall^4^)	PAM cl. 8	Low	High	Thrombospondin type 1 domain-containing protein (TGME49_209060); 7e-32
cgd1_3510	Q5CSA4 507 aa	TSP1 domain-containing protein TSP3	*No data available*	7–29	Many (ooocyst wall^4^, sporozoite^5^)	PAM cl. 8	Low	High	Thrombospondin type 1 domain-containing protein (TGME49_209060); 2e-24
cgd8_150	Q5CQ00 488 aa	CpTSP4 extracellular protein with signal peptide and 2 repeats of an apple domain followed by a TSP1 domain	Proteolysis Extracellular region Protein binding	5–24	None	*No data available*	Low	High	Thrombospondin type 1 domain-containing protein (TGME49_209060); 2e-19

^1^Localization was based on results reported in Guérin et al. (2023).

^2^Expression values were based on Mirhashemi et al. (2018).

^3^Proteomics data based on Snelling et al. (2007).

^4^Wang et al. (2022).

^5^Sanderson et al. (2008).

In parallel, we also explored the functional protein–protein interaction revealed by String to identify additional candidates for the rhomboid cleavage, and the interaction maps generated by the String analysis were reported as **Supplementary Material** (**Supplementary Figure 7**). We listed 11 proteins as potential substrates, considering only the proteins with a direct interaction with a rhomboid. It should be mentioned that 9 out of the 11 proteins of this list were attributed to interact with similar probability to each of the three *C. parvum* rhomboids.

Altogether, we listed 10 proteins as potential rhomboid substrates, with the following information (**Table 2**): gene ontology annotation, which indicates the presumptive molecular function, location, and/or involvement in a biological process. When available, we also included the MS/MS proteomic data and the localization in sporozoites by hiperLOPIT (Guérin et al., 2023). The approximate expression level was estimated based on mRNA in oocyst/sporozoite and intracellular stages (Mirhashemi et al., 2018).

All these proteins showed recognizable domains that indicated a putative function, but it was also evident that these *C. parvum* proteins could be phylogenetically classified in three orthologous groups based on the high similarity with specific proteins in *T. gondii* (last column on the right in **Table 2**).

Two of these proteins, namely, cgd2_1590 and cgd6_670, share a calcium-binding EGF domain and a similarity with two related but different *T. gondii* proteins, namely, CBDP2 and CBDP3 (Wang et al., 2023). Interestingly, these two proteins were independently identified by similarity and String analysis.

The larger phylogenetic group, five proteins in total, was instead related to the Sushi domain-containing protein (TGME49_223480). This domain, also named CCP for complement control protein or SCR for short consensus repeats, plays a complement inhibitor role (Gialeli et al., 2018). Importantly, four of these five proteins have been localized in micronemes of sporozoites, thereby sharing their localization with CpRom3 (Gao et al., 2021).

Finally, a small group of three proteins was highly related to a member of a TRAP protein family, namely, the thrombospondin type 1 domain-containing protein (TGME49_209060). Furthermore, two of them, precisely cgd1_3500 and cgd1_3510, have been mapped (Guérin et al., 2023) in the same spatial cluster (PAM cl. 8) and identified by multiple peptides in proteomics experiments both in the oocyst wall (Wang et al., 2022) and in sporozoites before the excystation (Sanderson et al., 2008) but not in excysted sporozoites as expected for early cleaved and secreted proteins. Therefore, these two TRAP proteins represented good candidates as precociously exposed adhesins to contact the receptors on the host cell surface.

## Discussion

This is the first comprehensive study on *C. parvum* rhomboids, and it is the first effort to compare these proteins in terms of phylogenesis, structural characteristics, and cell localization with other apicomplexan rhomboids. Moreover, this study also identifies the putative substrates of these membrane proteases. In *C. parvum*, three distinct rhomboids are co-expressed in the sporozoite stage but each with its own specific subcellular distribution. Two of them, CpRom1 and CpRom3, apparently disappear after the formation of PV, whereas CpRom2 persist in the PV after the host cell invasion. The differences in the localization as well as the persistence or the lack of it in intracellular stages indicate a different role in the parasite biology.

The genes for CpRom1 and CpRom2 consist of single exons, whereas the gene encoding for CpRom3 is composed of two exons. Genes composed of a single exon are the most common in *C. parvum*, since this species is characterized by a very compact genome (Abrahamsen et al., 2004). At the protein level, these three rhomboids have significant differences in size, even if the genetic similarities show that each of the three *C. parvum* rhomboids derives from a limited number of ancestral paralogues, probably two, that have branched out in the evolution of Apicomplexa (**Figure 2**). Remarkably, *C. parvum*, as well as other *Cryptosporidium* species, lacks a mitochondrial PARL-like rhomboid.

The alignments of *C. parvum* rhomboids with functional rhomboids of *P. falciparum* and *T. gondii* reveal the presence of two distinguishable clusters that can be classified based on a short amino acid consensus around the catalytic dyad. The first cluster includes CpRom1 and CpRom2 and comprises big rhomboids larger than 400 aa, and among them, CpRom1 is the largest rhomboid ever described (990 aa). This cluster shows a conserved triad GSS at the fourth TM and an AHXGG consensus at the sixth TM. Functionally, PfRom4, TgRom4, and TgRom5, which belong to this group, are involved in adhesins’ cleavage, suggesting a similar role for CpRom1 and CpRom2.

Differently, CpRom3 is related to a second cluster that includes PfRom1, PfRom3, TgRom1, TgRom2, and TgRom3. This group is characterized by a quadruple GAST amino acid sequence at the fourth TM and the GHIGG consensus at the sixth TM, and it is composed of smaller proteins of a maximum of 300 aa in length. Also, PfRom1, which is included in this cluster, has a demonstrated capacity to cleave adhesins (Baker et al., 2006) and probably plays a role in erythrocyte invasion (Singh et al., 2007). Differently, the roles of TgRom1, TgRom2, and TgRom3 are still uncertain even if their expression profiles are differentiated during the *T. gondii* life cycle (Brossier et al., 2005). Four rhomboids of this group, among them PfRom1 and CpRom3, also share a short consensus (FF or FPHF, (Sheiner et al., 2008) (**Supplementary Figure 2**) that allows the Golgi sorting of these proteins to the destination organelle (i.e., the micronemes for CpRom3). Based on the alignments and the TM domains, we have sketched the three *C. parvum* rhomboids and the PARL-related CmRom4 of *C. muris* for comparison (**Supplementary Figure 3**). In this scheme, CpRom1 is presented with an additional seventh TM domain, and in this updated version, the long N-terminus remains exposed on the external surface of the membrane as previously demonstrated (Bertuccini et al., 2024). Overall, all *C. parvum* rhomboids can be classified as mixed secretase with seven TM domains, a group in which functional apicomplexan rhomboids except for PARLs are included (Lemberg and Freeman, 2007).

As mentioned above, the similarity searches in *Cryptosporidium* spp. reveal that *C. parvum* lacks a PARL-like gene, although this subclass of rhomboids is instead present in *C. muris* and *C. andersoni.* It is worth mentioning that *C. muris*, besides being a PARL-like rhomboid, also conserves a functional mitochondrion (Uni et al., 1987). In addition, both *C. muris* and *C. andersoni* affect the stomach of their hosts and are thus indicated as “gastric” species. Differently, *C. parvum* and all the other species used for the genome comparison (**Figure 3**) are all parasites of the small intestine and can be considered as “intestinal” species. The loss of the single mitochondrion has been a distinctive step in the evolution of this genus since it establishes a remarkable difference among the *Cryptosporidium* species and even more with the other apicomplexans. Indeed, *C. parvum* and other intestinal species lack an aerobic metabolism, whereas this is active in *C. muris* (Uni et al., 1987) and *C. andersoni* (Liu et al., 2016). These observations raise questions about the role of aerobic metabolism and the mitochondrion in the gastric *Cryptosporidium* species. Could the mitochondrion as well as the aerobic metabolism simply be a residual inheritance from the ancestors of this taxonomic group? In line with this view, genetic evidence supports such hypothesis since *C. muris* is more closely related to the *Cryptosporidium* ancestor (Xiao et al., 1999). Alternatively, is it possible that gastric species have retained this metabolism to survive in the stomach? Regardless of these questions, the precise excision of PARL-like genes in intestinal *Cryptosporidium* species without the modification of the chromosomal syntenic structure is worthy of note.

The expression of the recombinant forms of *C. parvum* rhomboids in *E. coli* has been feasible in denaturing conditions, and the preparation of native proteins included in a cell membrane has been possible for 6h-CpRom2 and 6h-CpRom3 by means of expression in dedicated *E. coli* strains. Differently, the recombinant 6h-CpRom1 was always accumulated in insoluble inclusion bodies regardless of the *E. coli* strains used (**Supplementary Figure 1**). It is reasonable that the presence of multi-spanning hydrophobic TM domains, combined with the lack of a specific transit route to the bacterial membrane, makes it difficult to produce well-conformed *C. parvum* rhomboids in bacteria. Indeed, the fractionations of the lysates from the recombinant strains have demonstrated the prevalence of insoluble forms of these proteins in bacterial cells. This experiment has also shown that the allocation in the biological membrane is required to obtain integer forms of 6h-CpRom2 and 6h-CpRom3. Nevertheless, the purification of recombinant peptides derived from these rhomboids has been possible in denaturing conditions, thus allowing the production of specific mouse antisera.

CpRom2 and CpRom3 are expressed in intact oocysts and in excysted sporozoites similarly to CpRom1 (Bertuccini et al., 2024). Hence, the three *C. parvum* rhomboids are co-present at least in the early stage of the life cycle of the parasite. In the oocyst/sporozoite stage, CpRom2 has been identified as a unique band of approximately 70 kDa, and the difference from the expected molecular weight of 51 kDa may be due to post-translational modifications, such as glycosylation (**Figure 5A**). Indeed, the recombinant 6h-CpRom2 synthetized in *E. coli* and localized in the membrane fraction is approximately 50 kDa according to the expected molecular weight (**Supplementary Figure 1**). The amount of this protein remains unchanged from the oocyst to the excysted sporozoite, up to 1 h after the excystation starts (**Figure 5A**). Differently, CpRom3 showed three different forms that vary in quantity during the excystation process (**Figure 5B**). The three forms are represented by a larger band of approximately 65–70 kDa in size, an intermediate band of approximately 35 kDa, and a smaller one of roughly 30 kDa (**Figure 5B**). The 35-kDa band, which agrees with the expected molecular weight, appears only 30 min after the excystation and increases up to 1-h post-excystation. In contrast, the 30-kDa band appears abundant in the oocysts and decreases in the excysted sporozoites. However, the largest 70-kDa band is continuously present from the oocyst stage to the excysted sporozoites after 1 h. It is plausible that the largest 70-kDa band, which is approximately twice the expected molecular weight, may result from a paradoxical behavior of certain rhomboids that can assume a dimeric or even multimeric form in the presence of detergents (i.e., SDS) (Sampathkumar et al., 2012). Indeed, the recombinant 6h-CpRom3 showed a prevalent band of 35 kDa, accordingly with the expected molecular weight, when localized in the *E. coli* cell membrane (**Supplementary Figure 1**). Hypothetically, the largest band (70 kDa) and the smallest band observed in the inactive oocysts (30 kDa) could be the residual CpRom3 accumulated during the previous parasitic stages, whereas the 35-kDa band might represent the newly synthesized protein after the excystation. Overall, CpRom3 undergoes dynamic transformations at least during the early stage of the life cycle that modify forms and amounts of this protein.

We have then explored the localization of *C. parvum* rhomboids in the excysted sporozoites and showed that these proteins have a different spatial distribution.

Indeed, CpRom1 has a dual allocation: anterior to the nucleus and separately at the posterior end of the parasite. In the apical pole, the red labeling of this protein appears internal to the apical complex and not on the surface of the parasite, and the colocalization with the sporozoite antigen antiserum (Trasarti et al., 2007) that brightly labels in green the surface of sporozoites supports this interpretation (**Figures 6C, D**). Notably, this CpRom1 distribution is consistent with a reported localization on the inner membrane complex (IMC), still determined with a different method (Guérin et al., 2023). On the other hand, anti-CpRom1 antibodies also intensely stain in red a thickening at the posterior pole of the sporozoites (**Figure 6D**). This dual localization has been confirmed by immunolocalization at the ultrastructural level with electron microscopy, as the gold particles have been observed inside the apical complex in the area around micronemes as well as at the posterior pole of sporozoites (**Figures 6G–L**). A retrograde route taken by CpRom1 from the apical complex towards the posterior pole of the parasite would explain this peculiar distribution. The CpRom1 distribution recalls that of TgRom5, which is the rhomboid more closely related to CpRom1. Indeed, TgRom5 converges towards the posterior pole to remove adhesins that would interfere with the parasite ingress in the host cell (Brossier et al., 2005).

Differently, CpRom2 is exclusively distributed around the apical complex (**Figures 7A–G**), and the green labeling of this protein has always been observed anterior to the nucleus. Moreover, some of the non-permeabilized sporozoites show a brilliant green fluorescence on the apical surface, meaning that CpRom2, at least in part, is transferred to the apical surface of sporozoites early after the excystation.

The immunolocalization of *C. parvum* rhomboids in the intracellular stages in HCT8 cells has been limited to the first 48 h of infection; however, this experiment demonstrates that CpRom2 persists in the PV of the infected cells after the entry of the parasite. Hence, CpRom2 can be observed at intracellular stages of the parasite (**Figures 7H, L, N**). This suggests that, differently from CpRom1 and CpRom3, CpRom2 plays a role in the early internal stages of the life cycle of the parasite.

The localization of CpRom3 is further different from that of CpRom1 and CpRom2; in fact, this protein is distributed throughout the surface of the sporozoite from the anterior pole up to the posterior pole (**Figure 8**). The specific labeling also shows two points of thickening right on the extremities of the sporozoite (**Figure 8E**). This distribution is perfectly in accordance with previous subcellular localization that assigned this protein in the apical micronemes and on the pellicle of sporozoites (Gao et al., 2021). Overall, the localization in the micronemes and on the external membrane, as well as the high similarity with Pfrom1 (Baker et al., 2006), suggests that this rhomboid is probably involved in the adhesin cleavage along the entire sporozoite membrane.

The genetic manipulation of *C. parvum* to demonstrate functional interaction between a specific rhomboid and putative adhesins is still difficult; hence, the search for similarities with other apicomplexan adhesins is a feasible path to identify the putative targets of *C. parvum* rhomboids. The *in silico* analysis to identify plausible substrates for the *C. parvum* rhomboids identified various proteins with adhesive domains that most probably are involved in the first phases of the progression toward and the adhesion to the host enterocytes. Some of these proteins share domains and high phylogenetic relations with well-known adhesive proteins just described in other Apicomplexa. In this context, it is worth mentioning that the *C. parvum* genome did not reveal any protein related to the AMA proteins, which are instead present in *P. falciparum* and in *T. gondii* (data not shown) and are substrates of rhomboids in these parasites (Shen et al., 2014). On the other hand, all the identified *C. parvum* proteins are strictly related to orthologs in the *T. gondii* genome. Indeed, the two proteins with a calcium-binding EGF domain also identified by String, namely, cgd2_1590 and cgd6_670, are strictly related to CBDP2 and to CBDP3, which are two proteins of *T. gondii* with calcium-binding epidermal growth factor (EGF) domains. However, the role of these calcium-binding EGF domain-containing proteins such as CBDP2 and CBDP3 in *T. gondii* infection remains elusive (Wang et al., 2023).

Differently, the *C. parvum* proteins with a CCP domain may have an important function before the penetration of the sporozoite in the host cell. This search has identified a group of five proteins highly related to the Sushi domain (scr repeat) domain-containing protein (TGME49_223480) of *T. gondii*, and four of these proteins have been localized in micronemes (Guérin et al., 2023). Sushi domains are characteristic of mammalian proteins in the extracellular matrix and play a role in complement regulation (Gialeli et al., 2018). Among the parasites, proteins with the Sushi domain are present in Apicomplexa, and Sushi-provided proteins are also expressed in tissue larvae of nematodes (Hewitson et al., 2013). It is plausible that these parasitic proteins may play a role in reducing the action of the complement factors against the parasites.

Finally, a smaller group of three proteins is represented by *C. parvum* proteins related to the thrombospondin type 1 domain-containing protein (TGME49_209060) of *T. gondii*. This protein belongs to the TRAP family, and this type of multi-modular protein is known to be necessary for the invasion of the host cell (Morahan et al., 2009). TRAP proteins have multiple adhesive domains that allow adhesion to and gliding over the host cells (Paoletta and Wilkowsky, 2022). Two of these thrombospondin-related proteins, precisely cgd1_3500 and cgd1_3510, have been identified by multiple peptides in proteomics experiments both in the oocyst wall (Wang et al., 2022) and in sporozoites before the excystation (Sanderson et al., 2008), but not in excysted sporozoites (Snelling et al., 2007). Therefore, these adhesive proteins may represent precocious molecules exposed by the parasite to contact the receptors on the host cell surface. The identification of the *C. parvum* membrane proteins cleaved by rhomboids should be supported by dedicated proteomics experiments on the proteins secreted by sporozoites after the excystation and prior to the host cell invasion.

To conclude, this is the first comprehensive study on the three *C. parvum* rhomboids, and we have shown that these proteins are highly related to other Apicomplexa rhomboids. It has been demonstrated that these proteases, specifically in *P. falciparum* and *T. gondii*, play a crucial role in removing adhesive proteins from the cell surface, favoring a proper positioning and entry before and during the host cell invasion (Brossier et al., 2005; Howell et al., 2005; Buguliskis et al., 2010). In fact, genetically modified parasites in which some rhomboid functions were abolished have a strongly reduced infectious capacity (Shen et al., 2014). We have also identified a decade of protein candidates as rhomboid substrates, and these proteins are functionally related to *T. gondii* and *P. falciparum* proteins involved in the immunoevasion or in the attack of the host cells. Overall, this study identifies a group of sporozoite proteins, namely, rhomboids and their presumptive substrates, that can be targeted by therapy such as specific antibodies to block the parasite entry in intestinal enterocytes.

## Data Availability

The datasets presented in this study can be found in online repositories. The names of the repository/repositories and accession number(s) can be found in the article/**Supplementary Material**.
