# Supplementary material for: Exploring the repertoire of rhomboid proteases in Cryptosporidium parvum parasite: phylogenesis, structural motifs, and cellular localization in sporozoite cells
Source: Front Cell Infect Microbiol. 2026 Apr 7;16:1733450. doi: 10.3389/fcimb.2026.1733450 (PMC13095730; doi:10.3389/fcimb.2026.1733450)
Supplement: Supplementary file 2 [file DataSheet2.pdf]

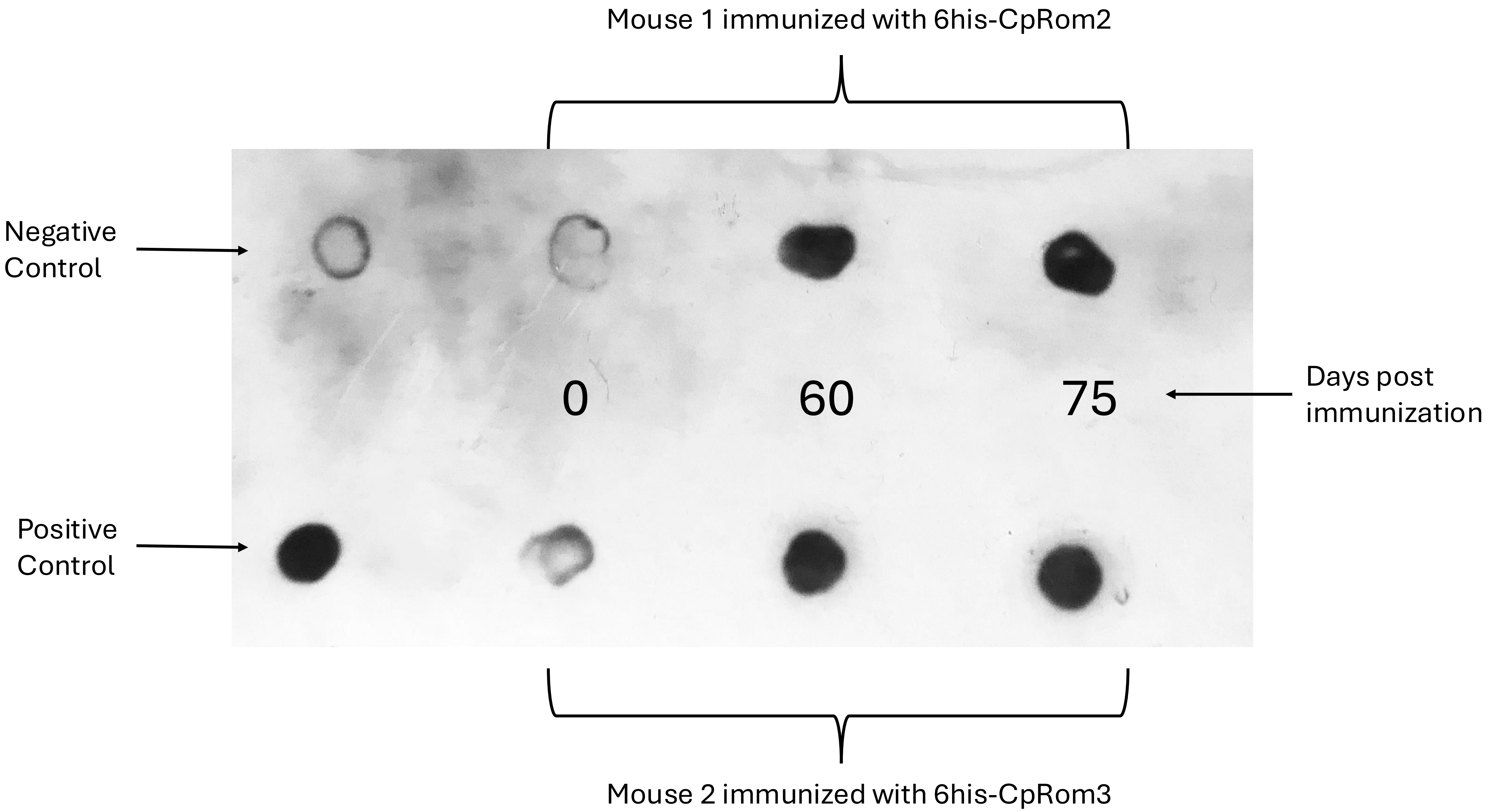
Mouse 1 immunized with 6his-CpRom2
Negative Control
Positive Control
0
60
75
Days post immunization
Mouse 2 immunized with 6his-CpRom3
