# Supplementary material for: Exploring the repertoire of rhomboid proteases in Cryptosporidium parvum parasite: phylogenesis, structural motifs, and cellular localization in sporozoite cells
Source: Front Cell Infect Microbiol. 2026 Apr 7;16:1733450. doi: 10.3389/fcimb.2026.1733450 (PMC13095730; doi:10.3389/fcimb.2026.1733450)
Supplement: Supplementary file 3 [file DataSheet3.pdf]

A

Alignments with homologs of CpRom1 and CpRom2 showing TM domains and catalytic sites

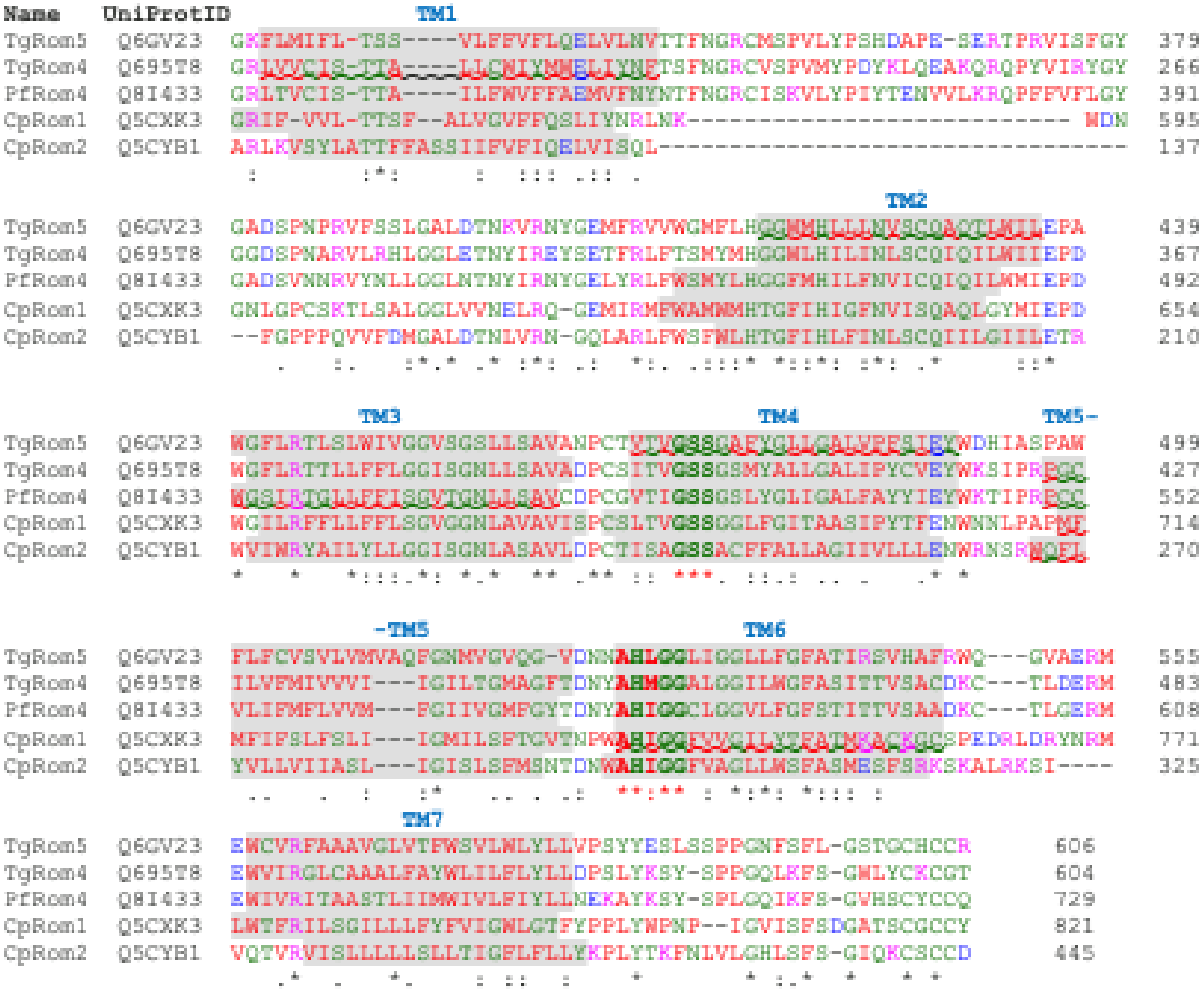

B

Alignments with homologs of CpRom3 showing TM domains and catalytic sites

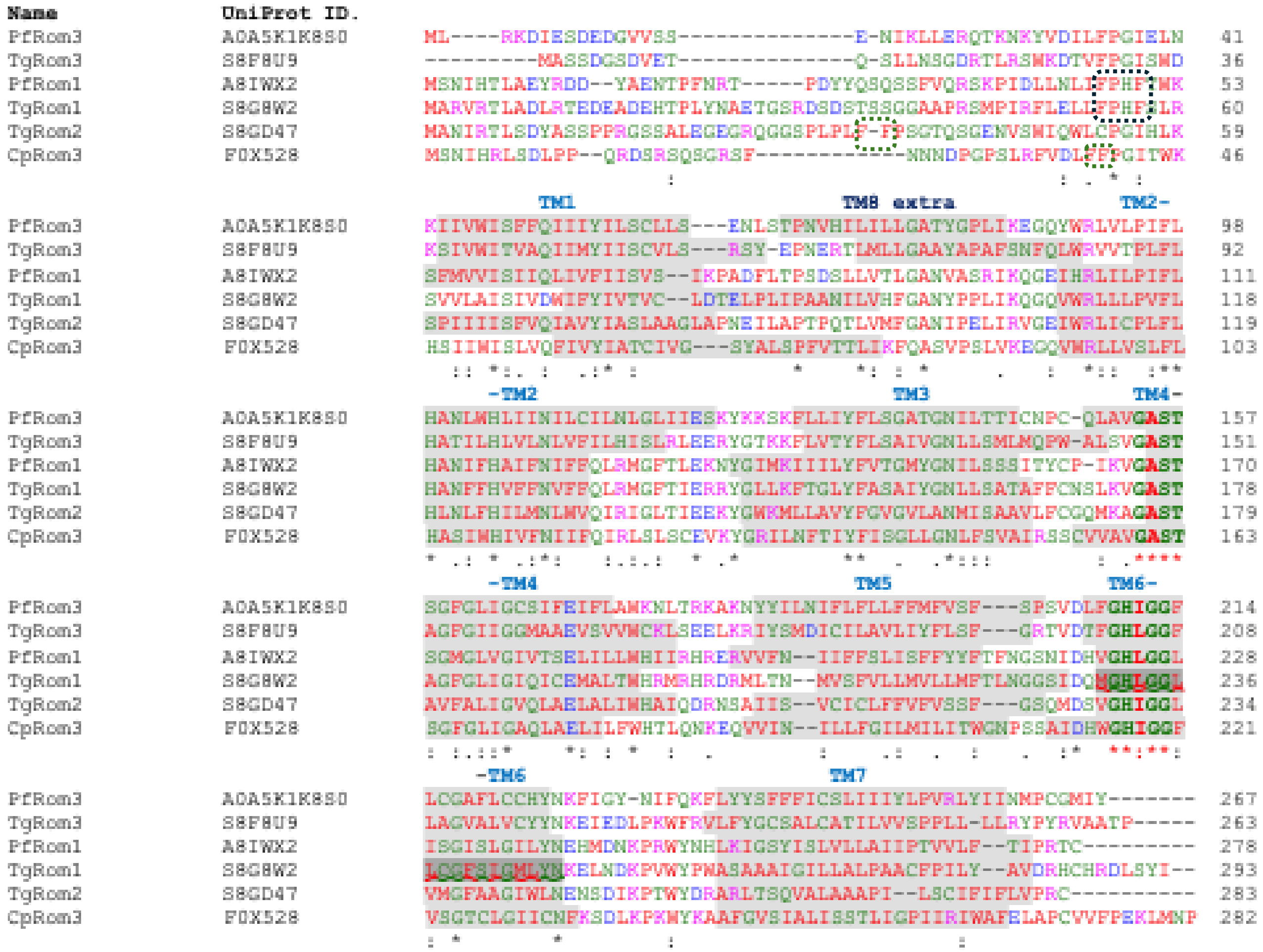

Supplementary Figure S2. A, Alignments with homologs of CpRom1 and CpRom2 showing TM domains and catalytic sites. Alignments were obtained with ClustalO. Gross part of the proteins at their N-terminus were eliminated to align the rhomboid domains of the proteins. B, TM domains and catalytic sites in CpRom3 aligned with similar proteins of Toxoplasma gondii and Plasmodium falciparum. Alignments were obtained with ClustalO. Shaded amino acids indicate predicted TM domains. Waved underlines indicate predicted TM domains obtained directly with TMHMM v.2. Wavy underscored TM domains were predicted based on the most similar overlapping sequence and verified by AlphaFold. Bold letters correspond to the catalytic dyad and the surrounding conserved amino acids. FPHF in blue dotted box indicate post-Golgi-sorting motif and FF in green dotted boxes indicate Golgi-targeting motif (Sheiner L et al., 2008).
