# Supplementary material for: Exploring the repertoire of rhomboid proteases in Cryptosporidium parvum parasite: phylogenesis, structural motifs, and cellular localization in sporozoite cells
Source: Front Cell Infect Microbiol. 2026 Apr 7;16:1733450. doi: 10.3389/fcimb.2026.1733450 (PMC13095730; doi:10.3389/fcimb.2026.1733450)
Supplement: Supplementary file 4 [file DataSheet4.pdf]

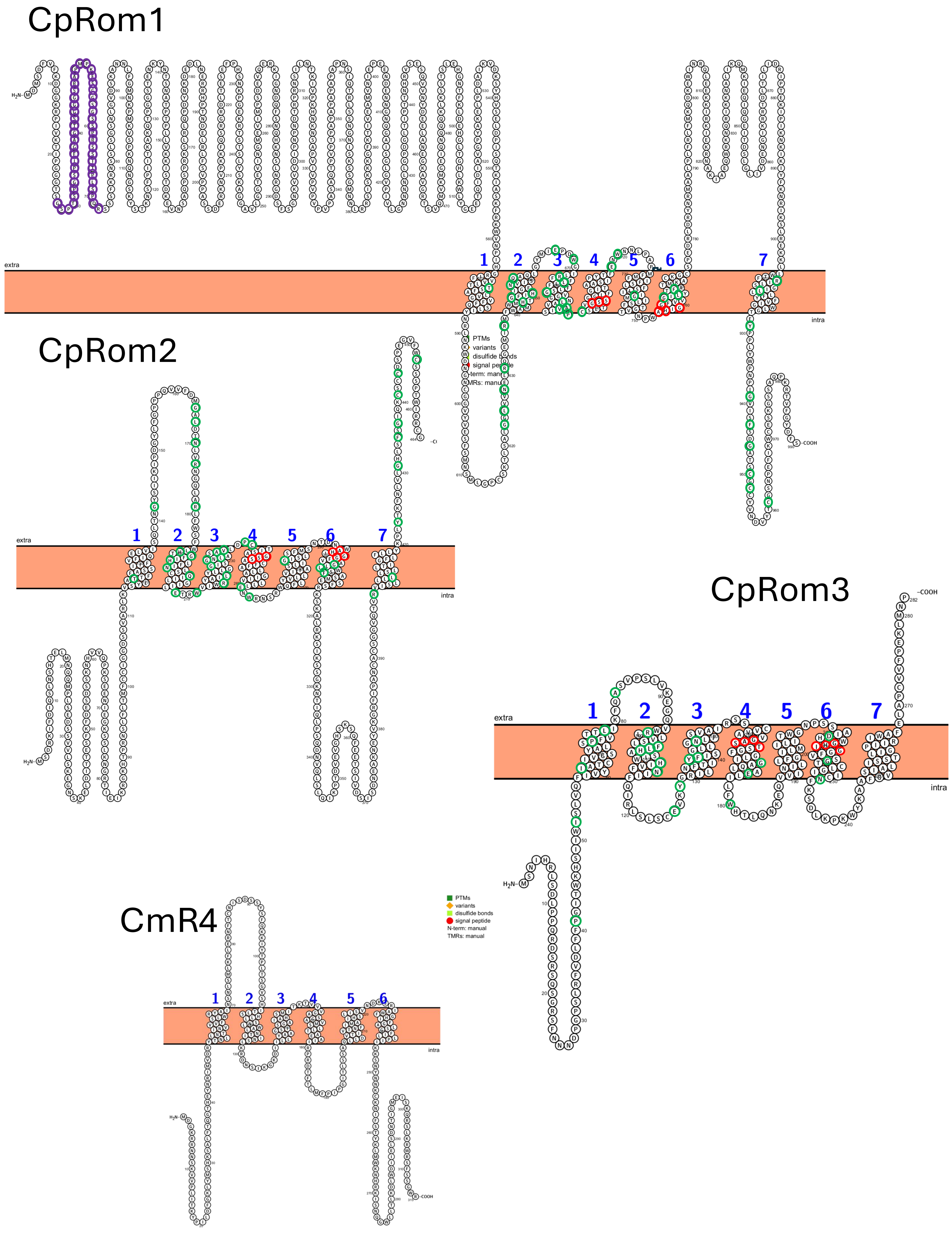


Supplementary figure S3. Bidimensional representation of the three *C. parvum* rhomboids and the PARL-like rhomboid CmR4 from *Cryptosporidium muris*. The circled amino acids represent the residues always conserved in the two clusters, namely one for CpRom1 and CpRom2 and the second for CpRom3, in red for the catalytic site and in green along the protein sequence (see in the text).
