# Supplementary figures and images for: Exploring the repertoire of rhomboid proteases in Cryptosporidium parvum parasite: phylogenesis, structural motifs, and cellular localization in sporozoite cells
Source: Front Cell Infect Microbiol. 2026 Apr 7;16:1733450. doi: 10.3389/fcimb.2026.1733450 (PMC13095730; doi:10.3389/fcimb.2026.1733450)

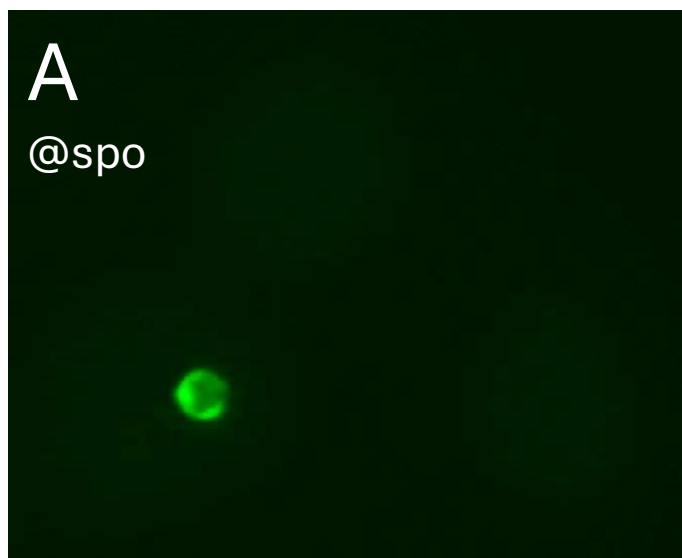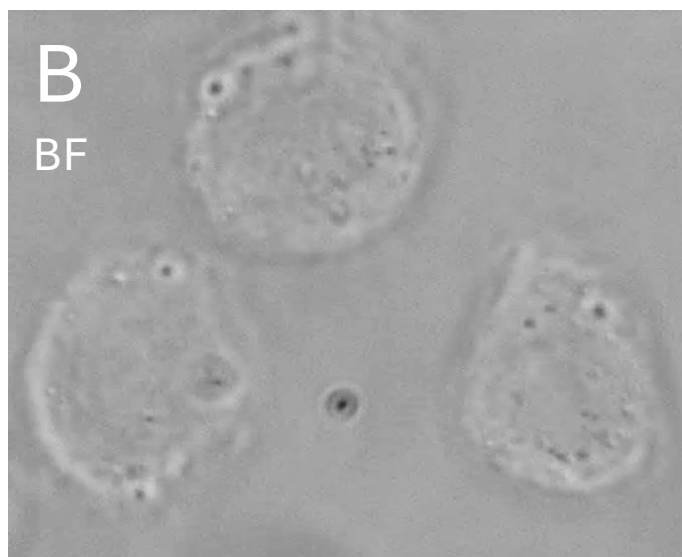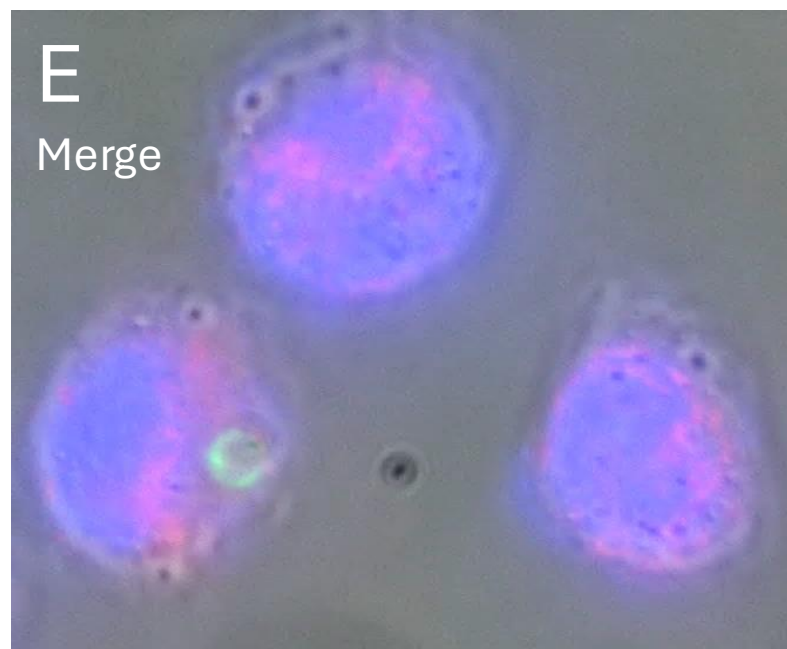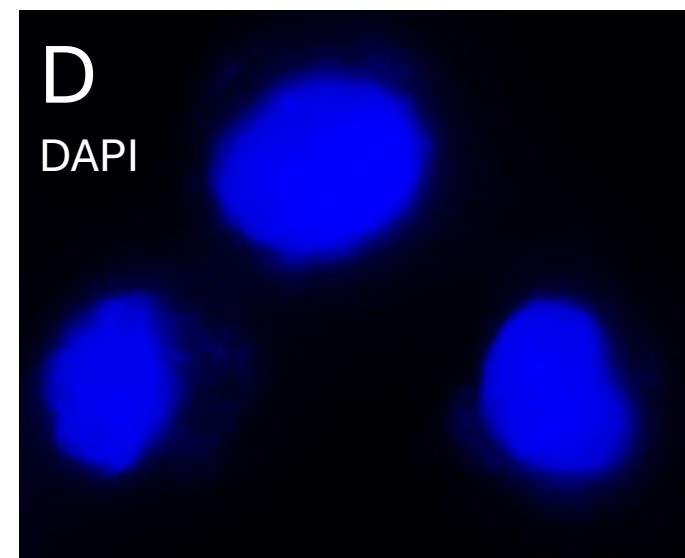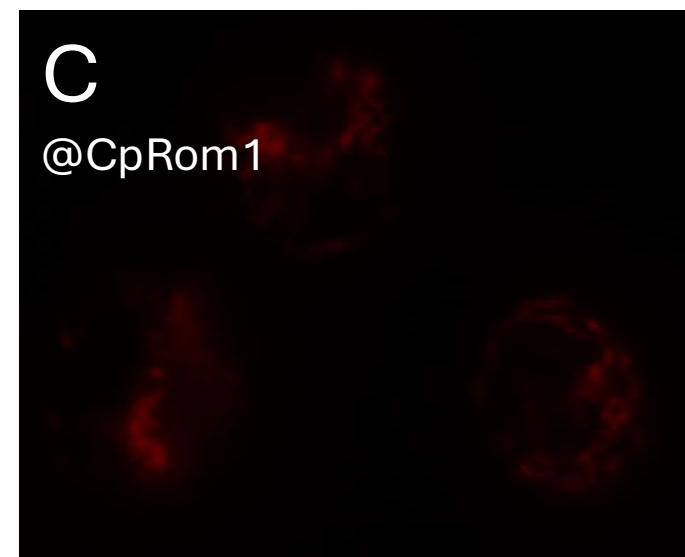

Supplement: Supplementary file 5 [file DataSheet5.pdf]

# 6h-CpRom1

Ruv3

Ly Sp In Mb

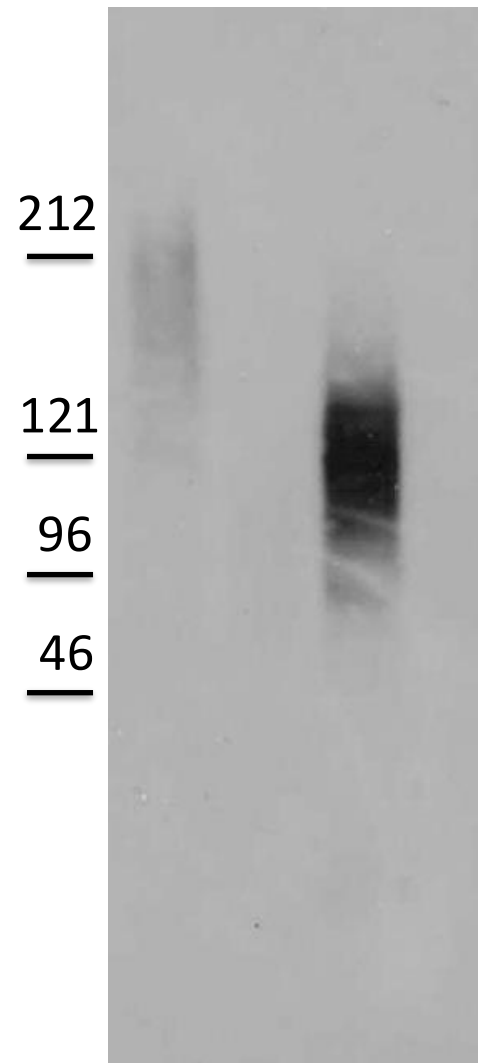

# 6h-CpRom2

M15

Ruv5

Ly Sp In Mb

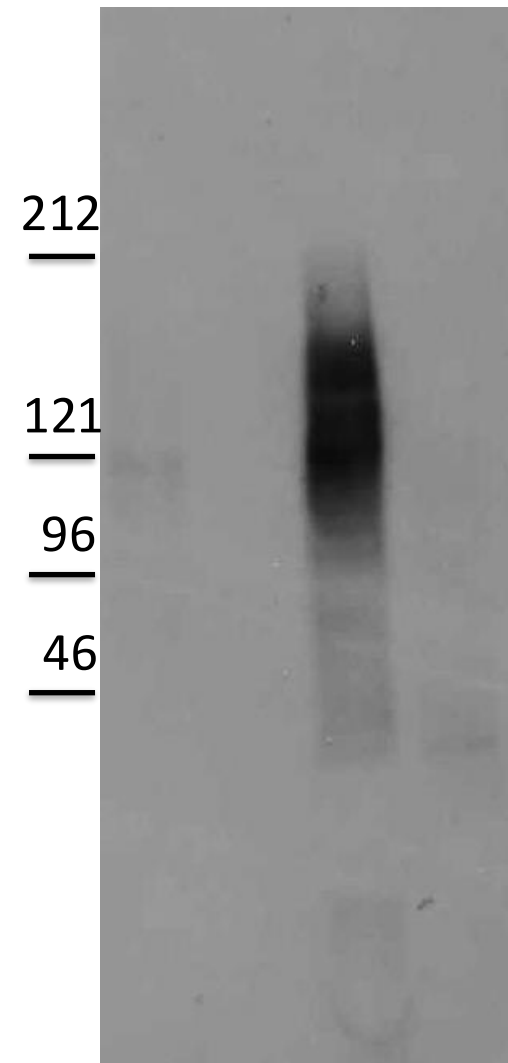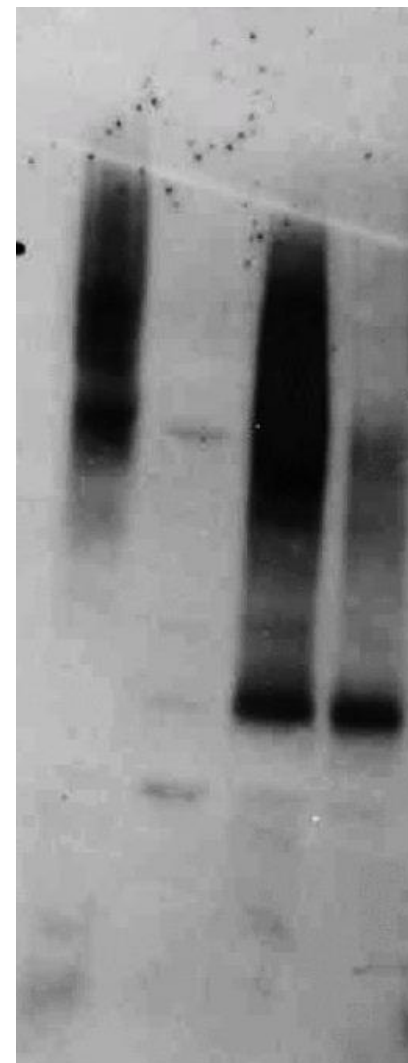

# 6h-CpRom3

M15

Ruv5

Ly Sp In Mb Ly Sp In Mb

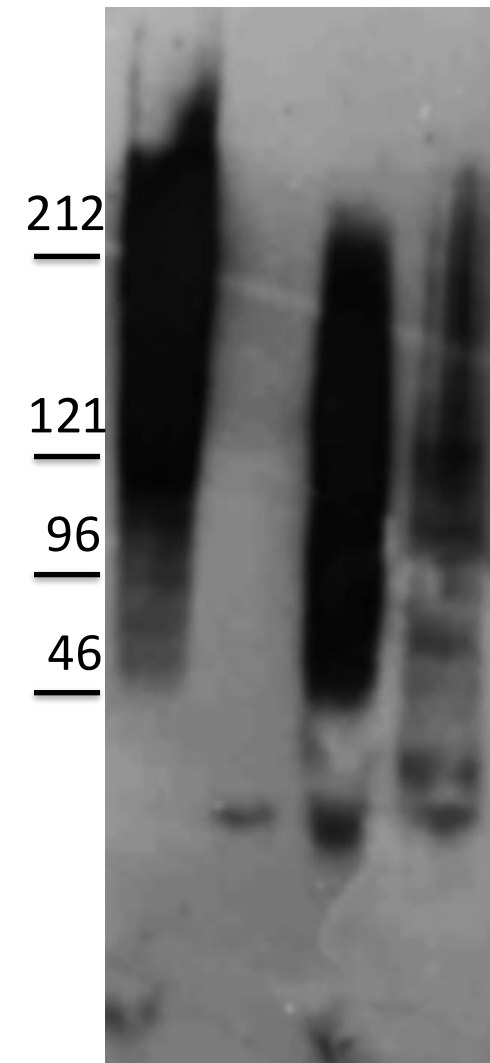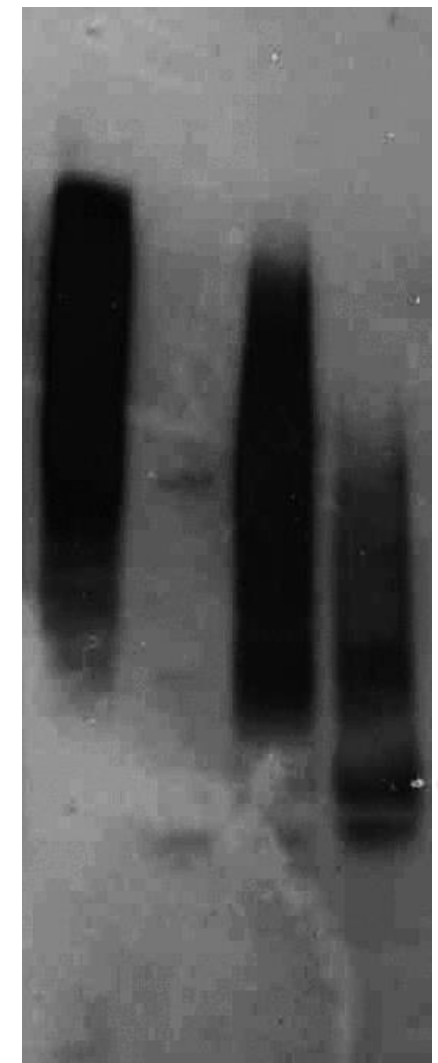

Supplement: Supplementary file 6 [file DataSheet6.pdf]

## C. parvum rhomboid constructs in expression vector pQE30

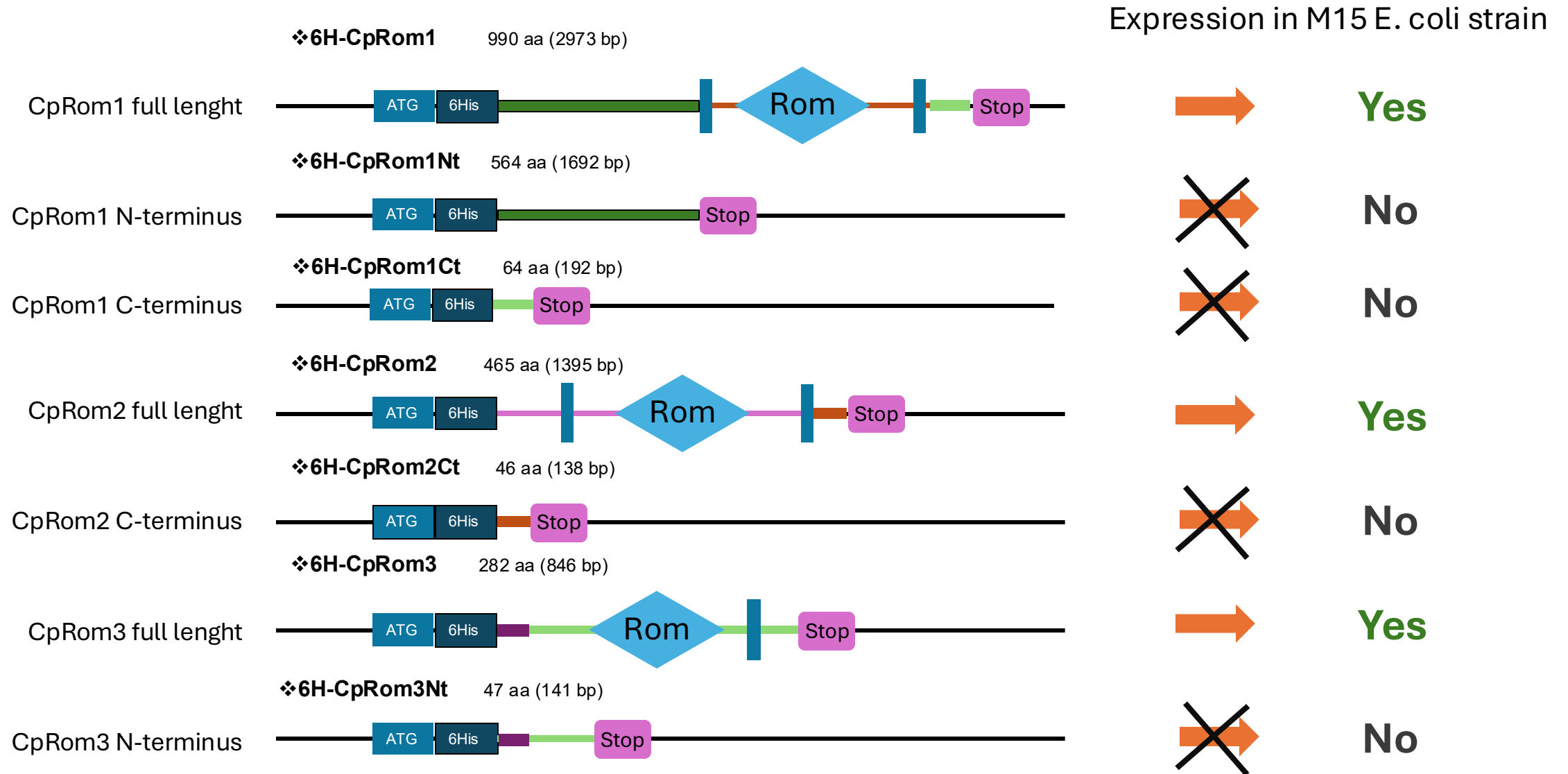

Supplement: Supplementary file 7 [file DataSheet7.pdf]
