# Supplementary material for: Exploring the repertoire of rhomboid proteases in Cryptosporidium parvum parasite: phylogenesis, structural motifs, and cellular localization in sporozoite cells
Source: Front Cell Infect Microbiol. 2026 Apr 7;16:1733450. doi: 10.3389/fcimb.2026.1733450 (PMC13095730; doi:10.3389/fcimb.2026.1733450)
Supplement: Supplementary file 8 [file DataSheet8.pdf]

Table 1, of oligonucletides used in this study.

Gene	Forward primers*	Reverse primers*	Function
CpRom1	ATGGATATGTCCGATTTTGTTTTC	TCAAGAAAAATCATATCCAAATAC	PCR on genomic DNA and RT-PCR on mRNA
CpRom1 Nterm	ATGGATATGTCCGATTTTGTTTTC	ATTAACAACTAAACCTCCAAGAGC	
CpRom1 Cterm	GGTTGTTCTCCTGAGGATAG	TCAAGAAAAATCATATCCAAATAC	
CpRom2	ATGTCTGACAGAAAGATTTTTG	TTATCCACATCTTCTAATCCATG	
CpRom3	CACAGACTTTCTGATTTACCTC	CCATACTTGACCCTCCTTAAC	
CpRom1	AACGAGCTCGATATGTCCGATTTTGTTTTCA	TCCCCCCGGGTCATCAAGAAAAATCATATCCAAATA	Cloning in expression vector
CpRom1 Nterm		TCCCCCCGGGTCAATGTATTGGATTAACCCATTTT	
CpRom1 Cterm	AACGAGCTCTTCTATCCTCCATTATATTGG	TCCCCCCGGGTCATCAAGAAAAATCATATCCAAATA	
CpRom2	AACGAGCTCTCTGACAGAAAGATTTTTGATAT	TCCCCCCGGGTTATCCACATCTTCTAATCCAT	
CpRom2 Cterm	AACGAGCTCTAAGCCATTGTACACTAAGTT		
CpRom3	CGCGGATCCTCAAATATACACAGACTTTCTG	TCCCCCCGGGTCAAGGATTCATAAGTTTCTCT	
CpRom3 Nterm		TCCCCCCGGGTTAACTATGTTTCCAAGTGATTCC	

*Underlined sequences represent restriction sites inserted for cloning.
