# Supplementary material for: Exploring the repertoire of rhomboid proteases in Cryptosporidium parvum parasite: phylogenesis, structural motifs, and cellular localization in sporozoite cells
Source: Front Cell Infect Microbiol. 2026 Apr 7;16:1733450. doi: 10.3389/fcimb.2026.1733450 (PMC13095730; doi:10.3389/fcimb.2026.1733450)
Supplement: Supplementary file 9 [file DataSheet9.pdf]

	UNIPROT	length	PFAM Peptidase S54 rhomboid		
CpRom1	Q5CXK3	990	633-769	567-587	638-657
CmRom1	B6ABI5	892	542-678	474-494	543-561
CpRom2	F0X3H6	464	175-309	114-131	184-206
CmRom2	B6ABU2	469	167-300	107-123	176-198
GnRom1	A0A023B066	350	198-333	139-158	207-228
TgRom5	Q6GV23	841	460-602	323-343	464-484
PfRom4	Q8I433	759	457-591	332-351	466-487
TgRom4	Q695T8	641	330-466	219-239	352-372
GnRom2	A0A023B0P3	448	90-229	40-58	98-122
PfRom3	A0A5K1K8S0	267	83-221	43-61	68-84
TgRom3	Q6IUY1	263	79-219	37-57	86-106
CpRom3	F0X528	282	92-230	49-71	94-116
CmRom3	B6AAF3	273	87-226	44-66	89-111
GnRom3	A0A023BBG2	276	94-231	50-71	96-118
TgRom2	Q695T9	283	106-244	62-82	114-134
PfRom1	A8IWX2	278	96-238	52-73	105-124
TgRom1	Q695U0	293	103-247	62-82	112-132
PfRom10	C6KT26	274	98-238	55-81	139-157
PfRom8	Q8ILY3	738	555-698	506-529	591-611
PfRom7	A0A5K1K991	340	170-337	188-205	211-231
TgRom6	Q2PP52	531	349-508	277-297	307-327
CmRom4	B6ADY7	315	92-246	50-69	150-173
PfRom6	A0A5K1K8X1	569	305-478	61-70	72-81
PfRom9	Q8I3V7	488	333-483	266-286	376-394

	UNIPROT	length	PFAM DER1 Derlin		
PfDer1	Q8IKV3	354	152-337	192-215	229-248
TgDer1	C9WWW6	589	303-493	353-376	388-411
PfDer1-2	Q8IJ82	263	12-205	19-42	51-74
PfDerlin-1	C7SP48	214	10-202	19-42	54-77
TgDer1ER2	C9WWW8	212	10-200	14-37	57-80

TM

669-691	697-715	727-746	904-925		
582-600	606-623	635-654	660-681	808-831	
213-233	239-258	270-288	294-314	400-419	
205-225	231-254	261-280	286-307	400-419	
240-257	263-282	294-311	317-341		
492-512	526-546	571-590	673-693		
521-541	553-572	578-599	681-703		
382-402	404-424	445-465	567-587		
134-152	158-177	189-206	212-234		
96-117	129-145	151-172	184-201	207-224	236-258
121-141	142-162	189-209	231-251		
128-151	157-180	189-208	214-233	245-266	
123-146	152-177	184-203	209-228	240-261	
130-152	158-178	190-206	218-236	248-270	
149-169	179-199	205-225	227-247	260-280	
136-154	166-190	196-214	221-239	254-274	
148-168	174-194	217-237	262-282		
169-189	195-214	221-238	250-272		
655-672	678-696	708-733			
264-286	292-311	318-337			
367-387	407-427	440-460	484-504		
184-207	226-245				
266-275	277-286	325-336	371-389	431-448	458-476
403-423	427-447	461-481			

TM

276-299	303-326	
458-481		
94-117	120-138	144-167
96-119	139-162	171-193
94-117	137-160	169-189
